# Estimating misclassification errors in the reporting of maternal mortality in national civil registration vital statistics systems: A Bayesian hierarchical bivariate random walk model to estimate sensitivity and specificity for multiple countries and years with missing data

**DOI:** 10.1002/sim.9335

**Published:** 2022-02-14

**Authors:** Emily Peterson, Doris Chou, Ann‐Beth Moller, Alison Gemmill, Lale Say, Leontine Alkema

**Affiliations:** ^1^ Department of Biostatistics and Bioinformatics Emory University Atlanta Georgia; ^2^ Department of Sexual and Reproductive Health and Research World Health Organization Geneva Switzerland; ^3^ Department of Population, Family, and Reproductive Health Johns Hopkins University Baltimore Maryland USA; ^4^ Department of Biostatistics and Epidemiology University of Massachusetts Amherst Amherst Massachusetts USA

**Keywords:** Bayesian hierarchical models, maternal mortality, misclassification

## Abstract

Civil registration vital statistics (CRVS) systems provide data on maternal mortality that can be used for monitoring trends and to inform policies and programs. However, CRVS maternal mortality data may be subject to substantial reporting errors due to misclassification of maternal deaths. Information on misclassification is available for selected countries and periods only. We developed a Bayesian hierarchical bivariate random walk model to estimate sensitivity and specificity for multiple populations and years and used the model to estimate misclassification errors in the reporting of maternal mortality in CRVS systems. The proposed Bayesian misclassification (BMis) model captures differences in sensitivity and specificity across populations and over time, allows for extrapolations to periods with missing data, and includes an exact likelihood function for data provided in aggregated form. Validation exercises using maternal mortality data suggest that BMis is reasonably well calibrated and improves upon the CRVS‐adjustment approach used until 2018 by the UN Maternal Mortality Inter‐Agency Group (UN‐MMEIG) to account for bias in CRVS data resulting from misclassification error. Since 2019, BMis is used by the UN‐MMEIG to account for misclassification errors when estimating maternal mortality using CRVS data.

## 1 INTRODUCTION

National civil vital registration (CRVS) systems record the number of deaths in a population, as well as the cause associated with each death using ICD coding.^1^ Under ideal circumstances, when all deaths are captured and all causes are accurately classified, CRVS systems provide perfect information on the number of deaths associated with a given course. However, cause‐specific deaths may be reported incorrectly if deaths are unregistered or misclassified, where misclassification of deaths refers to incorrect coding in vital registration systems, due either to error in the medical certification of cause of death or error in applying the correct ICD code. In the presence of under‐registration or misclassification, the estimation of cause‐specific mortality based on CRVS data needs to account for bias associated with reporting errors.

This article focuses on the estimation of misclassification errors, as summarized in sensitivity and specificity of reporting, for multiple populations and periods, in the presence of missing data. This work is motivated by the assessment of maternal mortality based on CRVS data for countries with CRVS systems. A maternal death is “the death of a woman whilst pregnant or within 42 days of termination of pregnancy, irrespective of the duration and site of the pregnancy, from any cause related to or aggravated by the pregnancy or its management but not from accidental or incidental causes.”^1^ The reporting of maternal mortality in CRVS systems is often subject to substantial misclassification errors.^2^, ^3^, ^4^ Prior work comparing the ratio of the proportion of deaths to women of reproductive age that are maternal (PM) as obtained from specialized studies—rigorous assessments of maternal mortality for a given country‐period—to CRVS‐based PMs found that these ratios are around 150%.^2^ Given that substantial misclassification errors may be present in CRVS recording of maternal deaths, accounting for such errors is necessary to obtain unbiased estimates of maternal mortality. Unfortunately, information on misclassification errors in CRVS systems is typically available for a limited number of years only. In addition, information may be available in aggregated form only, providing only the corrected and CRVS‐reported total number of maternal deaths, as opposed to the number of deaths that were misclassified.

We developed a Bayesian hierarchical misclassification model (referred to as the BMis model), which produces estimates of sensitivity and specificity for multiple population‐periods in presence of missing data. Our contributions to the statistical literature related to estimating sensitivity and specificity are twofold. First, we introduce a bivariate hierarchical temporal model to capture differences in sensitivity and specificity across populations and over time. This extends the work on bivariate modeling of sensitivity and specificity by Reitsma et al^5^ and the hierarchical modeling approach used by Chu and Cole^6^ to allow for extrapolations to periods with missing data and account for temporal autocorrelation in the estimation of sensitivity and specificity.^5^, ^6^ Second, we introduce an exact likelihood function for data provided in aggregated form.

The BMis model is used by the UN Maternal Mortality Inter‐Agency Group (UN‐MMEIG) to estimate and account for sensitivity and specificity in the reporting of maternal mortality from 1985 until 2017 for all WHO member states. The UN‐MMEIG publishes internationally comparable estimates of maternal mortality for UN reporting. The group uses a Bayesian hierarchical time series regression model (BMat) to estimate the proportion of maternal deaths out of all cause deaths (PM).^3^, ^7^, ^8^ In BMat, estimates of the PM are produced based on the available input data for the respective country‐period, taking account of data quality issues in reporting. In prior rounds of constructing estimates, the UN‐MMEIG adjusted CRVS data based on simplifying assumptions, that is, keeping constant the ratio of bias‐corrected to CRVS‐reported PM in country‐periods with missing data on misclassification. We show that BMis improves upon the prior UN‐MMEIG approach in validation exercises.

This article is organized as follows: Section 2 introduces the data available to inform estimation of the extent of incorrect reporting. Section 3 introduces a Bayesian model to estimate the extend of misclassification in the reporting of maternal deaths in CRVS systems. The estimation is based on summarizing misclassification in terms of sensitivity and specificity, and modeling these two indicators for all country‐years with CRVS data using a bivariate hierarchical random walk model. Finally, we present findings and the validation exercises.

## 2 DATA

### 2.1 CRVS data on maternal mortality

The WHO Mortality Database maintains data from CRVS systems.^9^ CRVS systems collect information based on death certification data, which provides ICD coded certified causes of death. Further information regarding CRVS systems and how they vary across countries can be found in Mikkelsen et al.^10^ For each country‐year c,t, this database provides the number of maternal deaths reported in the CRVS, denoted by $y_{c,t}^{\left(\text{mat}\right)}$ and the number of deaths to women aged 15 to 49 reported in the CRVS, denoted as $y_{c,t}^{\left(\text{crvs}\right)}$, referred to as the CRVS envelope.

In this study, we focus on assessing misclassification errors among deaths reported within CRVS systems. Deaths reported within CRVS systems are all deaths to women of reproductive ages for systems that capture all deaths, and a subset of such deaths for incomplete systems that do not capture all deaths. Completeness of the reporting of deaths into the CRVS system was assessed by comparing CRVS‐reported deaths to WHO estimates of deaths to women of reproductive age, obtained from life tables for WHO Member States.^7^, ^11^ In this study, we focus on data from country‐years for which CRVS completeness is either deemed complete, or for which we have specialized studies data referring to the deaths as reported in the CRVS.

### 2.2 Specialized studies data

In the context of this study, we define specialized studies as those studies that provide accurate information on the number of maternal deaths among CRVS‐reported deaths of women of reproductive age. Specialized studies include a review of cause of death for all deaths to women of reproductive age registered in the CRVS system. These studies may be carried out independently of CRVS‐reported data or based on the checking of CRVS‐reported deaths.

We constructed a data base with information on maternal mortality from specialized studies. Specialized studies were obtained through (1) a literature review, (2) the UN MMEIG 2015 maternal mortality data base,^3^ and (3) information provided by countries based on a follow‐up survey, sent to countries in response to discussions with the Pan‐American Health Organization (PAHO), and during country consultation. Details on the literature review and inclusion criteria are provided in Supplementary Appendix Section S6.2.

### 2.3 Data on misclassification errors in CRVS reporting of maternal mortality

Misclassification errors in CRVS reporting of maternal mortality refers to the inaccurate attribution of a maternal cause of death, due to error in the medical certification of cause of death, and/or error in applying the correct code. Misclassification errors occur in the form of either false negative (F−) or false positive (F+) maternal deaths. False negative maternal deaths refer to deaths that are of a maternal cause but not reported as such in the CRVS while false positives refer to non‐maternal deaths that are reported as maternal in the CRVS.

**FIGURE 1:**
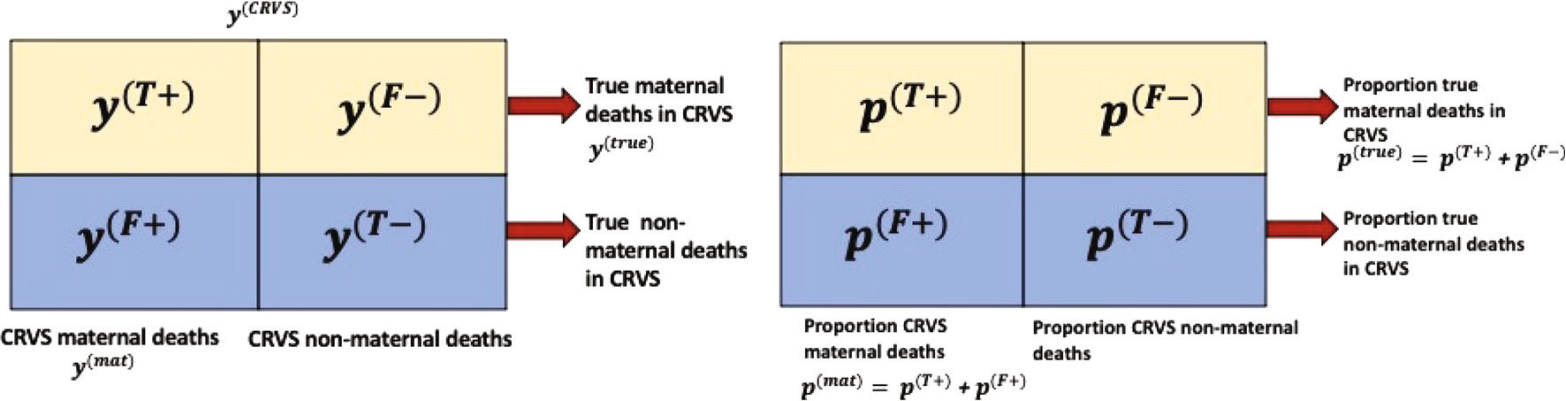
Diagram of breakdown of CRVS reporting of deaths to women of reproductive age for a country‐year, by CRVS‐assigned maternal cause (columns) and true maternal cause (rows). (Left) $y^{\left(T+\right)}$ and $y^{\left(F-\right)}$ deaths refer to maternal deaths that are correctly registered as maternal deaths, and incorrectly registered as non‐maternal deaths, respectively. Similarly, $y^{\left(F^{+}\right)}$ and $y^{\left(T-\right)}$ maternal deaths refers to non‐maternal deaths that are incorrectly registered as maternal deaths, and correctly registered as non‐maternal deaths, respectively. (Right) Corresponding observed proportions denoted by *p* referring to counts $y^{\left(T+\right)}$, $y^{\left(F-\right)}$, $y^{\left(F^{+}\right)}$, and $y^{\left(T-\right)}$ out of total CRVS‐reported deaths to women of reproductive age $y^{\left(\text{CRVS}\right)}$

The diagram in Figure 1 illustrates misclassification errors in CRVS systems by breaking down total deaths to women of reproductive age by CRVS‐reporting status (columns) and true maternal cause (rows). For country‐year c,t, the total number of false negative and false positive maternal deaths is denoted as $y_{c,t}^{\left(F-\right)}$ and $y_{c,t}^{\left(F+\right)}$, respectively. From the individual categories in Figure 1, cumulative totals are calculated summing across rows and columns, that is, CRVS‐reported maternal deaths, denoted by $y^{\left(\text{mat}\right)}$, are the sum of $y_{c,t}^{\left(T+\right)}$ and $y_{c,t}^{\left(F+\right)}$, whereas, the true number of maternal deaths within the CRVS, $y_{c,t}^{\left(\text{true}\right)}$, is the sum of $y_{c,t}^{\left(T+\right)}$ and $y^{\left(F-\right)}$. For a complete list of definitions, refer to Table S4 in Supplementary Appendix S6.1.

Information on CRVS misclassification errors was obtained from comparing information from CRVS‐reported deaths to data from specialized studies that refer to the same envelope of deaths. Data availability is summarized in Table 1. We have information from studies on 204 unique country‐periods (observations) in 32 countries. Reported information varied across observations. While some studies reported a detailed breakdown of false positive and/or false negative maternal deaths, the majority of studies reported only the confirmed total number of maternal deaths for a given country‐period (147 observations, 26 countries). Information on both false negative and false positive breakdowns was available for 18 observations (4 countries). Most studies with breakdown information solely reported on false negative breakdowns, 39 observations from 5 countries.

**TABLE 1:** Overview of data available from combining specialized studies and CRVS data

Reported counts	# of observations	# of countries
True maternal in CRVS only	147	26
F− and F+	18	24
F− only	39	5
Total	204	32

## 3 METHODS

### 3.1 Notation and definitions of parameters

We refer to observed quantities using lower‐case Roman letters, with $y_{c,t}^{\left(b\right)}$ and $p_{c,t}^{\left(b\right)}$ referring to observed counts and proportions of deaths in the CRVS for country *c* in year *t* in category *b*, respectively, with b∈B={T+,T−,F+,F−}, see Figure 1. CRVS‐reported maternal deaths are denoted by $y_{c,t}^{\left(\text{mat}\right)}=y_{c,t}^{\left(T+\right)}+y_{c,t}^{\left(F+\right)}$, CRVS‐reported total deaths are denoted by $y_{c,t}^{\left(\text{CRVS}\right)}$, and the true number of maternal deaths in the CRVS is denoted by $y_{c,t}^{\left(\text{true}\right)}=y_{c,t}^{\left(T+\right)}+y_{c,t}^{\left(F-\right)}$.

**FIGURE 2:**
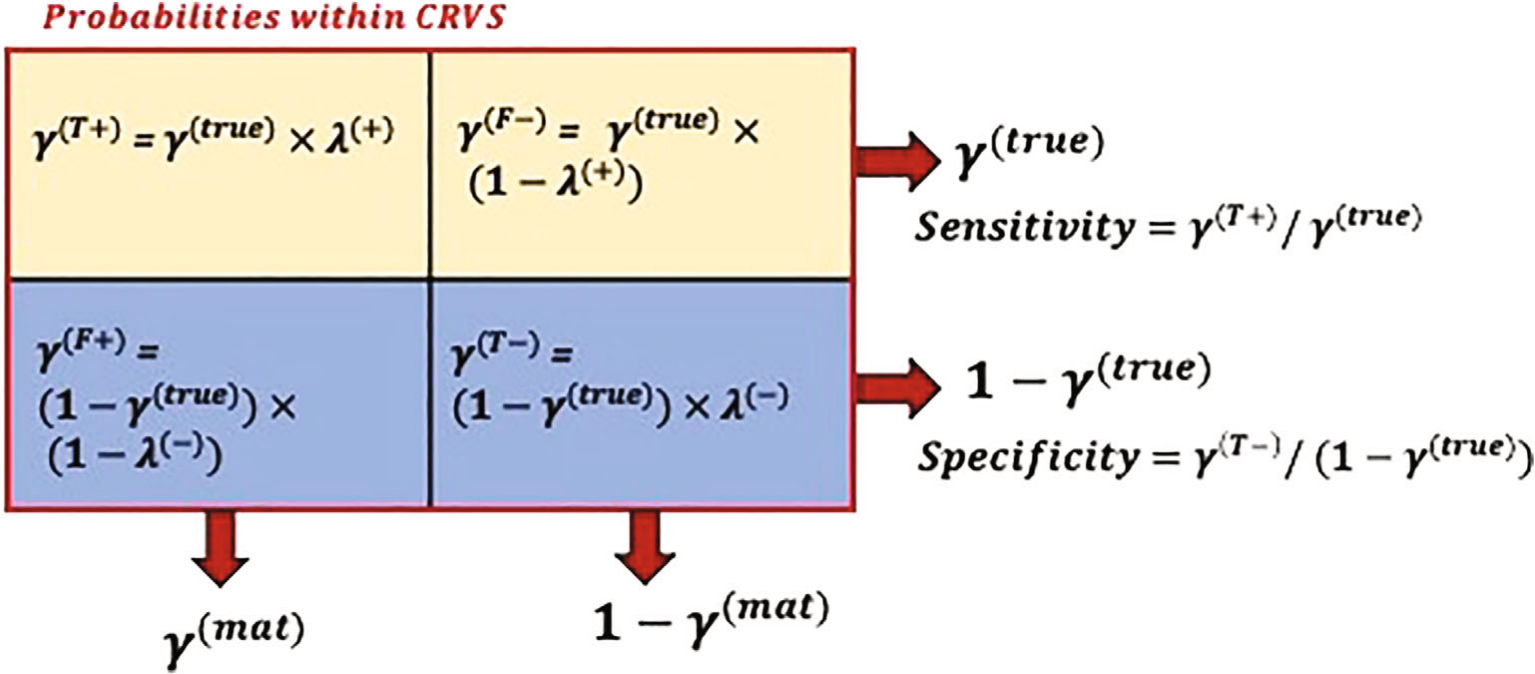
Diagram of breakdown of probabilities within CRVS systems for a given country‐year, by CRVS‐reporting status (columns) and true maternal cause (rows), where $\gamma^{\left(b\right)}$ refers to the probability of a CRVS‐reported death being in category b∈B={T+,T−,F+,F−}. The sum of $\gamma^{\left(T+\right)}$ and $\gamma^{\left(F-\right)}$ probabilities equal the probability of a true maternal deaths within CRVS $\left(\gamma^{\left(\text{true}\right)}\right)$. Similarly, $\gamma^{\left(F^{+}\right)}$ and $\gamma^{\left(T-\right)}$ equal the probability of a reported maternal death within CRVS $\left(\gamma^{\left(\text{mat}\right)}\right)$. The four probabilities are parameterized into metrics of $\text{sensitivity}=\frac{\gamma^{\left(T+\right)}}{\gamma^{\left(\text{true}\right)}}$ and $\text{specificity}=\frac{\gamma^{\left(T-\right)}}{1-\gamma^{\left(\text{true}\right)}}$

We refer to unknown parameters using lower‐case Greek letters. With the same notation for sub‐ and superscripts as introduced above for *y*, we let $\gamma_{c,t}^{\left(b\right)}$ refer to the probability that a death that is reported in the CRVS is classified into category *b*, such that $\sum_{b\in B}\gamma_{c,t}^{\left(b\right)}=1$, see Figure 2. For example, $\gamma^{\left(F-\right)}$ refers to the probability of a false negative maternal death within the CRVS system, and $\gamma^{\left(F+\right)}$ refers to the probability of a false positive maternal death within the CRVS system, respectively. With this definition of counts *y* and probabilities γ, it follows that,

(1) $$\begin{array}{ll} y_{c,t}|y_{c,t}^{\text{(CRVS)}},\gamma_{c,t}∼\text{Multinom}\left(y_{c,t}^{\text{(CRVS)}},\gamma_{c,t}\right), & \end{array}$$

where (leaving out subscripts (c,t) for improve readability):

$$\begin{array}{ll} y & =\left(y^{\text{(T−)}},y^{\text{(T+)}},y^{\left(F-\right)},y^{\left(F+\right)}\right),\;\\ \gamma & =\left(\gamma^{\text{(T−)}},\gamma^{\text{(T+)}},\gamma^{\left(F-\right)},\gamma^{\left(F+\right)}\right).\; \end{array}$$

We define sensitivity of CRVS reporting of maternal mortality as the probability that a maternal death in the CRVS is classified as such: $\lambda^{\left(+\right)}=\gamma_{c,t}^{\left(T+\right)}/\gamma_{c,t}^{\left(\text{true}\right)}$, and specificity as a non‐maternal death classified as such: $\lambda_{c,t}^{\left(-\right)}=\gamma_{c,t}^{\left(T-\right)}/(1-\gamma_{c,t}^{\left(\text{true}\right)})$. Based on the definitions of sensitivity $\lambda_{c,t}^{\left(+\right)}$ and specificity $\lambda_{c,t}^{\left(-\right)}$, we can write the cell‐specific probabilities $\left(\gamma_{c,t}^{\left(T+\right)},\gamma_{c,t}^{\left(F-\right)},\gamma_{c,t}^{\left(F+\right)},\gamma_{c,t}^{\left(T-\right)}\right)$ as a function of both measures and the true probability of a maternal death $\gamma_{c,t}^{\left(\text{true}\right)}$, see Figure 2. The relation between the CRVS‐reported probability of a maternal death $\gamma_{c,t}^{\left(\text{mat}\right)}$ and the true probability $\gamma_{c,t}^{\left(\text{true}\right)}$ is as follows:

(2) $$\gamma^{\text{(mat)}}=\lambda^{\left(+\right)}\gamma^{\text{(true)}}+\left(1-\lambda^{\left(-\right)}\right)\left(1-\gamma^{\text{(true)}}\right).$$

### 3.2 Objective and summary of modeling approach

We aim to estimate sensitivity $\lambda_{c,t}^{\left(+\right)}$ and specificity $\lambda_{c,t}^{\left(-\right)}$ in the reporting of maternal mortality for *all* country‐years with CRVS data, including country‐years for which no specialized study data are available, such that CRVS data can be used to inform the estimation of maternal mortality while accounting for CRVS misclassification errors.

We developed a bivariate hierarchical random walk model for estimating sensitivity and specificity for all country‐years. The process model for sensitivity and specificity is introduced in Section 3.3. We obtained estimates of sensitivity and specificity for maternal mortality reporting in CRVS systems using all available data for model fitting, including data on the total number of maternal deaths only. Model fitting is explained in Section 3.4, including the specification of the likelihood function.

### 3.3 Bivariate hierarchical random walk model for sensitivity and specificity

We developed a bivariate hierarchical random walk model to estimate sensitivity $\lambda_{c,t}^{\left(+\right)}$ and specificity $\lambda_{c,t}^{\left(-\right)}$ for all countries *c* with CRVS data for some year(s) *t*. Estimates of sensitivity and specificity were constrained to be within 0 and 1 using probit‐transformations.

The model set‐up used is a bivariate hierarchical random walk process on probit‐transformed sensitivity (se) and specificity (sp). In brief, we model the country‐year specific probit‐transformed se and sp as the sum of country‐specific average levels over the period of interest (1985‐2017) plus a linear combination of first order differences. The model captures correlation in sensitivity and specificity, which may arise based on efforts to improve the classification of maternal deaths. Specifically, if a CRVS system attempts to capture more true maternal deaths then there will be an increase in sensitivity, that is, more true maternal deaths are captured due to increase in classified maternal deaths. However, there may also be a decrease in specificity, that is, more non‐maternal deaths will be classified as maternal, which increases the rate of false positive maternal deaths. Therefore, accounting for this correlation is important for estimating misclassification parameters, that is, see Chu et al.^5^, ^6^ Details on the model specification are provided in the remainder of this section.

Let probit‐transformed se and sp be denoted by $\eta_{c,t}^{\left(+\right)}=\Phi (\lambda_{c,t}^{\left(+\right)})$ and $\eta_{c,t}^{\left(-\right)}=\Phi (\lambda_{c,t}^{\left(-\right)})$, respectively, where Φ() denotes the probit‐transform. With the definition of country‐specific averages $\eta_{c}^{\left(\right)}=\frac{1}{T}\sum \eta_{c,t}^{\left(\right)}$ and first‐order differences $\xi_{c,t}^{\left(\right)}=\eta_{c,t}^{\left(\right)}-\eta_{c,t-1}^{\left(\right)}$, we can write the η's as linear combinations of the average levels and first order differences,

(3) $$\begin{array}{ll} \eta_{c,1:T}^{\left(+\right)} & =\eta_{c}^{\left(+\right)}+D\cdot \xi_{c,1:T-1}^{\left(+\right)},\\ \eta_{c,1:T}^{\left(-\right)} & =\eta_{c}^{\left(-\right)}+D\cdot \xi_{c,1:T-1}^{\left(-\right)}, \end{array}$$

where matrix $D=W^{\prime }\times {\left(W\cdot W^{\prime }\right)}^{-1}$, with

$$W=\left[\begin{array}{cccccc} -\;1 & 1 & 0 & 0 & \ldots & 0\\ 0 & -1 & 1 & 0 & \ldots & 0\\ \vdots & \ddots & \ddots & \ddots & \ddots & \vdots \\ 0 & 0 & 0 & \ldots & -1 & 1 \end{array}\right].$$

We assume a hierarchical bivariate distribution for country‐specific levels of average probit‐transformed sensitivity and specificity given by:

(4) $$\begin{array}{ll} \left(\begin{array}{c} \eta_{c}^{\left(+\right)}\\ \eta_{c}^{\left(-\right)} \end{array}\right) & ∼N_{2}\left(\left[\begin{array}{c} \eta_{\text{global}}^{\left(+\right)}\\ \eta_{\text{global}}^{\left(-\right)} \end{array}\right],\left[\begin{array}{cc} \sigma_{}^{{\left(+\right)}^{2}} & \rho \cdot \sigma_{}^{\left(+\right)}\cdot \sigma_{}^{\left(-\right)}\\ \rho \cdot \sigma_{}^{\left(+\right)}\cdot \sigma_{}^{\left(-\right)} & \sigma_{}^{{\left(-\right)}^{2}} \end{array}\right]\right), \end{array}$$

in which the country‐specific levels of transformed sensitivity and specificity $\left(\eta_{c}^{\left(+\right)},\eta_{c}^{\left(-\right)}\right)$ are distributed bivariate normal centered on their respective global levels $\left(\eta_{\text{global}}^{\left(+\right)},\eta_{\text{global}}^{\left(-\right)}\right)$. The variance and correlation terms, associated with global levels of probit‐transformed sensitivity and specificity, are denoted by $\sigma^{\left(+\right)},\sigma^{\left(-\right)}$, and ρ.

The first‐order differences, $\xi_{c,t}$ are modeled with a zero‐mean bivariate normal given by:

(5) $$\begin{array}{ll} \left(\begin{array}{c} \xi_{c,t}^{\left(+\right)}\\ \xi_{c,t}^{\left(-\right)} \end{array}\right) & ∼N_{2}\left(\left[\begin{array}{c} 0\\ 0 \end{array}\right],\left[\begin{array}{cc} \delta^{{\left(+\right)}^{2}} & \phi \cdot \delta^{\left(+\right)}\cdot \delta^{\left(-\right)}\\ \phi \cdot \delta^{\left(+\right)}\cdot \delta^{\left(-\right)} & \delta^{{\left(-\right)}^{2}} \end{array}\right]\right). \end{array}$$

We assigned uniform prior distributions to the inverse‐probit transformed global mean values of probit‐transformed sensitivity and specificity. Specifically, we define $\lambda_{\text{global}}^{\text{()}}=\Phi^{-1}\left(\eta_{\text{global}}^{\left(\right)}\right)$, or equivalently, $\eta_{\text{global}}^{\text{()}}=\Phi \left(\lambda_{\text{global}}^{\text{()}}\right)$, and assign uniform priors to the $\lambda_{\text{global}}^{\text{()}}$'s:

$$\begin{array}{lcr} \lambda_{\text{global}}^{\left(+\right)} & ∼ & \text{Unif}(0,1),\\ \lambda_{\text{global}}^{\left(-\right)} & ∼ & \text{Unif}(0,1). \end{array}$$

Prior distributions for the correlation and standard deviations of the random walk are given below, using vague half‐normal distributions for variance parameters:^12^

(6) $$\begin{array}{ll} \phi & ∼\text{Unif}(-1,1), \end{array}$$

(7) $$\begin{array}{ll} \rho & ∼\text{Unif}(-1,1), \end{array}$$

(8) $$\begin{array}{ll} \sigma^{\left(\right)} & ∼N_{T(0,\infty )}(0,1), \end{array}$$

(9) $$\begin{array}{ll} \delta^{\left(\right)} & ∼N_{T(0,\infty )}(0,1), \end{array}$$

where $N_{T(0,\infty )}(0,1)$ denotes a half‐normal distribution (a truncated normal distribution with lower bound at 0).

We explored the use of indicators gross domestic product (GDP), the general fertility rate (GFR), the proportion of ill‐defined causes, CRVS completeness, and ICD coding (ICD10 or earlier) as possible covariates to inform estimates of sensitivity and specificity. However, exploratory analyses suggested no substantially meaningful relations. Covariates were not included in the final model (see Supplementary Appendix Section S6.3 for exploratory plots shown in Figures S7 and S8.

### 3.4 Model fitting

#### 3.4.1 Process model

For each country‐year with data, we define the four unknown CRVS‐based probabilities $\gamma_{c,t}^{\text{(b)}}$ in terms of the two misclassification parameters $\lambda_{c,t}^{\left(+\right)}$ and $\lambda_{c,t}^{\left(-\right)}$, and the true CRVS‐based probability of a maternal death $\gamma_{c,t}^{\text{(true)}}$ as follows:

(10) $$\begin{array}{ll} \gamma_{c,t}^{\text{(T+)}} & =\lambda_{c,t}^{\left(+\right)}\cdot \gamma_{c,t}^{\text{(true)}},\\ \gamma_{c,t}^{\left(F-\right)} & =\gamma_{c,t}^{\text{(true)}}-\gamma_{c,t}^{\text{(T+)}},\\ \gamma_{c,t}^{\text{(T‐)}} & =\lambda_{c,t}^{\left(-\right)}\cdot \left(1-\gamma_{c,t}^{\text{(true)}}\right),\\ \gamma_{c,t}^{\left(F+\right)} & =\left(1-\gamma_{c,t}^{\text{(true)}}\right)-\gamma_{c,t}^{\text{(T−)}}. \end{array}$$

Misclassification parameters are defined through the bivariate hierarchical random model on $\lambda_{c,t}^{\left(+\right)}$ and $\lambda_{c,t}^{\left(-\right)}$, as explained in the previous section. We use vague independent priors on $\gamma_{c,t}^{\text{(true)}}$:

$$\gamma_{c,t}^{\text{(true)}}∼U(0.0001,1).$$

#### 3.4.2 Likelihood function

We define $z_{i}^{\text{(b)}}$ to refer to the observed death count for category *b* in study i=1,…,n. We let c[i] denote the country to which study *i* refers. The start calendar year for study *i* is indexed by t1[i], its end year by t2[i], and its observation period midyear is given by t[i]. As per earlier notation, let $y_{c,t}^{\text{(b)}}$ denote the number of deaths in category *b*, for country *c* in year *t*, such that we can write $z_{i}^{\text{(b)}}=\sum_{t=t1[i]}^{t2[i]}y_{c[i],t}^{\text{(b)}}$.

We relate multinomial study counts $z_{i}=\left(z_{i}^{\text{(T−)}},z_{i}^{\text{(T+)}},z_{i}^{\left(F-\right)},z_{i}^{\left(F+\right)}\right)$ to the corresponding within CRVS probabilities $\gamma_{c,t}=\left(\gamma_{c,t}^{\text{(T−)}},\gamma_{c,t}^{\text{(T+)}},\gamma_{c,t}^{\left(F-\right)},\gamma_{c,t}^{\left(F+\right)}\right)$, assuming a multinomial data generation process as follows:

(11) $$\begin{array}{ll} z_{i}|z_{i}^{\text{(CRVS)}},\gamma_{c[i],t[i]} & ∼\text{Multinom}\left(z_{i}^{\text{(CRVS)}},\gamma_{c[i],t[i]}\right), \end{array}$$

with $z_{i}^{\text{(CRVS)}}=\sum_{b}z_{i}^{\text{(b)}}$, and unknown probability vector $\gamma_{c,t}=\left(\gamma_{c,t}^{\text{(T−)}},\gamma_{c,t}^{\text{(T+)}},\gamma_{c,t}^{\left(F-\right)},\gamma_{c,t}^{\left(F+\right)}\right)$.

The corresponding density for $z_{i}|z_{i}^{\left(\text{CRVS}\right)},\gamma_{c[i],t[i]}$ is given by,

(12) $$\begin{array}{ll} p_{z}(z_{i}|z_{i}^{\left(\text{CRVS}\right)},\gamma_{c[i],t[i]})=\frac{z_{i}^{\left(\text{CRVS}\right)}!}{\prod_{b}z_{i}^{\left(b\right)}!}\underset{b}{\prod }{\gamma_{c[i],t[i]}^{\left(b\right)}}^{z_{i}^{\left(b\right)}}. & \end{array}$$

Study reported data fall into two categories of data reporting. The first category consists of studies which report a specific set of non‐overlapping death counts $\left(z_{i}^{\text{(T−)}},z_{i}^{\text{(T+)}},z_{i}^{\left(F-\right)},z_{i}^{\left(F+\right)}\right)$ or some non‐overlapping subset or aggregation of these counts. The second category consists of studies which report overlapping counts, that is, studies reporting true maternal counts $z_{i}^{\text{(true)}}=z_{i}^{\text{(T+)}}+z_{i}^{\left(F-\right)}$, which overlaps with the CRVS‐reported maternal deaths for the corresponding country‐period, $z_{i}^{\text{(mat)}}=z_{i}^{\text{(T+)}}+z_{i}^{\left(F+\right)}$. We give a simple illustration of data in both categories and details on the corresponding likelihood function in the Supplementary Appendix, see Section S6.4.1. The likelihood functions are summarized in the remainder of this section.

For studies that report on a specific set of non‐overlapping categories, that is, the number of false positive maternal deaths and/or the number of true positive maternal deaths, the corresponding likelihood function was obtained directly using the multinomial data generating process in Equation (11). Specifically, the likelihood function for studies that report F+ and F− deaths, all four categories, is given directly by $p_{z}\left(z|z_{i}^{\text{(CRVS)}},\gamma_{c[i],t[i]}\right)$, where $p_{z}$ refers to the multinomial density function for the four CRVS‐based categories from Equation (11). For studies that report $\left(z_{i}^{\left(F-\right)},z_{i}^{\left(T-\right)},z_{i}^{\left(\text{mat}\right)}\right)$, the likelihood function follows from aggregating the relevant non‐overlapping categories in the multinomial:

$$\begin{array}{ll} \left(z_{i}^{\left(F-\right)},z_{i}^{\left(T-\right)},z_{i}^{\left(\text{mat}\right)}\right)|z_{i}^{\text{(CRVS)}},\gamma_{c[i],t[i]}∼\text{Multinom}\left(z_{i}^{\text{(CRVS)}},\left(\gamma_{c[i],t[i]}^{\left(F-\right)},\gamma_{c[i],t[i]}^{\text{(T‐)}},\gamma_{c[i],t[i]}^{\text{(mat)}}\right)\right). & \end{array}$$

The second category of studies consists of the majority of studies, which only reported information on the number of true maternal deaths within the CRVS (see Table 1). For these studies, data are given by $d_{i}=(z_{i}^{\text{(true)}},z_{i}^{\text{(mat)}})$ and the study‐reported count of maternal deaths, $z_{i}^{\text{(true)}}=z_{i}^{\text{(T+)}}+z_{i}^{\left(F-\right)}$, overlaps with the CRVS‐reported maternal deaths for the corresponding country‐period, $z_{i}^{\text{(mat)}}=z_{i}^{\text{(T+)}}+z_{i}^{\left(F+\right)}$. Details on the corresponding likelihood function are given in Section S6.4.2. In brief, for each study period with information on overlapping categories, we obtained the exact likelihood function for the available death counts by summing over multinomial densities evaluated at each unique combination that satisfied the observed set of counts. We obtain the complete set of unique combinations denoted by ${\tilde{z}}_{i}^{\left(s\right)}=\left({\tilde{z}}_{i}^{{\left(T+\right)}^{\left(s\right)}},{\tilde{z}}_{i}^{{\left(F+\right)}^{\left(s\right)}},{\tilde{z}}_{i}^{{\left(T-\right)}^{\left(s\right)}},{\tilde{z}}_{i}^{{\left(F-\right)}^{\left(s\right)}}\right)$ for s=1,…,S[i], which satisfies the observed marginals, given by,

(13) $$\begin{array}{ll} {\tilde{z}}_{i}^{{\left(mat\right)}^{\left(s\right)}} & ={\tilde{z}}_{i}^{{\left(T+\right)}^{\left(s\right)}}+{\tilde{z}}_{i}^{{\left(F+\right)}^{\left(s\right)}}=z_{i}^{\left(\text{mat}\right)}, \end{array}$$

(14) $$\begin{array}{ll} {\tilde{z}}_{i}^{\left(true)(s\right)} & ={\tilde{z}}_{i}^{{\left(T+\right)}^{\left(s\right)}}+{\tilde{z}}_{i}^{{\left(F-\right)}^{\left(s\right)}}=z_{i}^{\left(\text{true}\right)}. \end{array}$$

The likelihood function for data $d_{i}$ is given by:

(15) $$\begin{array}{ll} p(d_{i}|\gamma_{c[i],t[i]})=\overset{S[i]}{\underset{s=1}{\sum }}p({\tilde{z}}_{i}^{\left(s\right)}|\gamma_{c[i],t[i]})\cdot k_{i}^{\left(s\right)}, & \end{array}$$

with density $p({\tilde{z}}_{i}^{\left(s\right)}|\gamma_{c[i],t[i]})$ given in Equation (11). Indicator function $k_{i}^{\left(s\right)}$ is added to exclude combinations with negligible probability of being the true combination: $k_{i}^{\left(s\right)}=0$ for combination *s* with negligible probability of being the true combination, and $k_{i}^{\left(s\right)}=1$ otherwise. This indicator function was added to improve computational efficiency when fitting the model to studies with large numbers of unique combinations. Exclusions are carried out for combinations that are based on sensitivity less than 0.97, accounting for stochastic uncertainty. The indicator function is defined as follows:

(16) $$\begin{array}{ll} k_{i}^{\left(s\right)}=1\left({\tilde{z}}_{i}^{{\text{(T−)}}^{\left(s\right)}}>q_{i}^{\left(0.97\right)}\right), & \end{array}$$

where $q_{i}^{\left(0.97\right)}$ refers to the 2.5th percentile of a Binomial distribution with sample size $z_{i}^{\text{(CRVS)}}-z_{i}^{\text{(true)}}$ and probability 0.97. For further information on exclusion of combinations with negligible probability, see Appendix Section S6.4.2.1.

#### 3.4.3 Computation

A Markov chain Monte Carlo (MCMC) algorithm was employed to sample from the posterior distribution of the parameters with the use of the software *JAGS*.^13^, ^14^ Ten parallel chains were run with a total of 40 000 iterations in each chain. Of these, the first of 10 000 iterations in each chain were discarded as burn‐in and every 20th iteration after was retained. The resulting chains contained 1500 samples each, with a total of 15 000 posterior samples. Standard diagnostic checks (using trace plots and Gelman and Rubin diagnostics) were used to check convergence.^15^

### 3.5 Comparison of estimates of misclassification bias in CRVS‐based PM between the BMis model and the UN MMEIG 2015 approach

#### 3.5.1 UN‐MMEIG2015 approach

The BMat model, which estimates the proportion of maternal deaths out of all‐cause deaths, uses CRVS adjustment factors, denoted $\Omega_{c,t}$ for country *c* and year *t*, to correct for bias in CRVS‐based reported PM associated with misclassification of maternal deaths.^8^ In the UN MMEIG 2015 approach, the CRVS adjustment factor was summarized using the ratio of the observed PM as reported in specialized studies to the PM reported in the CRVS, resulting in point estimate ${\hat{\Omega }}_{c,t}^{\text{(UN‐MMEIG2015)}}$. The adjustment ratios were obtained for all country‐years with CRVS data and used in a CRVS bias‐adjustment preprocessing step.^8^ For countries with specialized studies for a subset of country‐years only, linear interpolation was used to obtain adjustments in years in between observed adjustments. For forward extrapolation, the CRVS adjustment was kept constant at the level of the most recent observed CRVS adjustment. For backward extrapolations in countries with studies, the CRVS adjustment was assumed to increase or decrease linearly to the same global adjustment factor of 1.5 in 5 years. The uncertainty of the adjustment was expert‐based: the standard deviation of the log‐transformed adjustment was set equal to the variability associated with $\Omega_{c,t}$, defined as follows:

$$\log(\Omega_{c,t}^{\text{(UN‐MMEIG2015)}})∼N\left(\log({\hat{\Omega }}_{c,t}^{\text{(UN‐MMEIG2015)}}),0.25^{2}\right).$$

#### 3.5.2 BMis approach

Our approach to assess bias and associated CRVS adjustments differs from the UN MMEIG 2015 approach; the CRVS adjustment factor in the BMis model is obtained from MCMC samples of sensitivity and specificity and varies with the true PM. Specifically, the model‐based sample estimate of the CRVS adjustment factor $\Omega_{c,t}^{{\left(\text{BMis}\right)}^{\left(s\right)}}$ for country *c* in year *t*, *s*th sample, with complete CRVS is given by:

(17) $$\begin{array}{ll} \Omega_{c,t}^{{\left(\text{BMis}\right)}^{\left(s\right)}}=\frac{\gamma_{c,t}^{\text{(true)}}}{\lambda_{c,t}^{{\text{(+)}}^{\left(s\right)}}\cdot \gamma_{c,t}^{\text{(true)}}+\left(1-\lambda_{c,t}^{{\left(-\right)}^{\left(s\right)}}\right)\cdot \left(1-\gamma_{c,t}^{\text{(true)}}\right)}, & \end{array}$$

in which $\lambda_{c,t}^{\left(s\right)}$ refers to the *s*th sample estimates of sensitivity and specificity from BMis, and the true PM $\gamma_{c,t}^{\left(\text{true}\right)}$ is estimated within the BMat model. In using MCMC samples, we propagate the uncertainty associated with sensitivity and specificity into the derived samples for the CRVS adjustment factor.

### 3.6 Model validation

**FIGURE 3:**
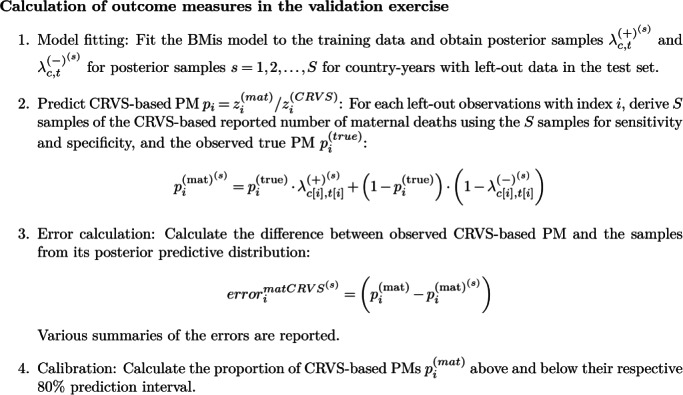
Overview of calculation of errors and coverage of prediction intervals in out‐of‐sample validation exercises

We carried out model validation to check the performance of the proposed approach in terms of predicting the relative bias associated with CRVS‐reported PMs and compared it to the UN‐MMEIG (2015) approach. Model performance was assessed through the two out‐of‐sample validation exercises. In the first exercise (repeated 20 times), 20% of the observations were left out at random to form a training data set. In the second exercise, we left out the observation corresponding to the most recent study period in each country. In each exercise, we fitted the BMis model to the training set and obtained posterior samples for sensitivity and specificity in the country‐years with left‐out specialized studies. We combined samples of sensitivity and specificity with the reported study‐based PMs to obtain samples of predicted CRVS‐based PMs. We summarized the difference between the model‐based predictions and the reported CRVS‐based PM in terms of error, that is, the difference between the observed CRVS‐based PM and its point estimate, and coverage of 80% prediction intervals. The procedure is described in detail in Figure 3.

The UN MMEIG 2015 approach was validated using the same training and test sets and results were compared between the two approaches. For the UN‐MMEIG approach, observed CRVS adjustment factors were calculated based on the training set, and interpolation and extrapolation was carried out as described in Section 3.5.

## 4 RESULTS

### 4.1 Validation results

The BMis model performs well in out‐of‐sample validation exercises, see Table 2. Median and relative errors are small in both exercises, and absolute errors are around 8% when predicting the CRVS‐based PM. The model is well calibrated, the coverage of the 80% prediction intervals is around 80%, with around 10% falling below (above) the lower (upper) bounds.

**TABLE 2:** Validation results

Error in CRVS‐PM								
			Median errors		Relative error (%)		Outside 80% PI	
Validation	Model	# left‐out obs	ME	MAE	MRE	MARE	Prop below	Prop above
Leave‐out 20% at random	BMis model	43	−0.00006	0.0004	−0.6	4.6	0.13	0.10
	UN MMEIG 2015	43	−0.00010	0.0009	−1.8	15.9	0.08	0.05
Leave‐out last observation	BMis model	20	0.0001	0.0007	3.2	6.1	0.10	0.10
	UN MMEIG 2015	20	−0.0003	0.0010	−4.0	14.4	0.10	0.10

*Note*: The outcome measures are: median error (ME), median absolute error (MAE), relative error (MRE), absolute relative error (MARE), as well as the % of left‐out observations below and above their respective 80% prediction intervals (PI) based on the training set. For the leave‐out 20% exercise, reported errors and coverage summarize within‐country mean errors and coverage.

The BMis model improves upon the UN‐MMEIG 2015 approach in terms of prediction error: mean errors and mean absolute errors are smaller in magnitude in the BMis model as compared to the UN‐MMEIG 2015 approach. For example, the median absolute relative error (MARE) decreases from 15.9% to 4.6% between the UN‐MMEIG 2015 and the BMis approach when predicting the observations left out at random. Similarly, in the leave‐out last observation validation, there is a reduction in median absolute relative error from 14.4% to 6.1% between the old and new model.

### 4.2 Global findings

Table 3 lists the posterior estimates of the hyperparameters and their respective 80% credible intervals (CI). Global estimates of sensitivity and specificity were estimated to be 0.593 with 80%CI given by (0.523,0.659) and 0.9995 (0.9992, 0.9997), respectively. The correlation of changes in sensitivity and specificity in countries over time was estimated to be negative at −0.737 (−0.370, 0.096). Correlation between average levels of country‐specific sensitivity and specificity was estimated to be uncertain with CI ranging from −0.409 to 0.786.

**TABLE 3:** Posterior estimates of global parameters; median estimate (50%) and lower (10%) and upper (90%) bounds of 80% credible intervals

	10%	50%	90%
Global sensitivity $\lambda_{\text{global}}^{\left(+\right)}$	0.523	0.593	0.659
Global specificity $\lambda_{\text{global}}^{\left(-\right)}$	0.9992	0.9995	0.9997
Correlation (global) ρ	−0.409	0.307	0.786
SD (global) sensitivity $\sigma^{\left(+\right)}$	0.450	0.599	0.806
SD (global) specificity $\sigma^{\left(-\right)}$	0.0344	0.157	0.326
Correlation (first‐order differences) ϕ	−0.737	−0.370	0.096
SD (first‐order differences) sensitivity $\delta^{\left(+\right)}$	0.095	0.117	0.146
SD (first‐order differences) specificity $\delta^{\left(-\right)}$	0.099	0.131	0.162

**FIGURE 4:**
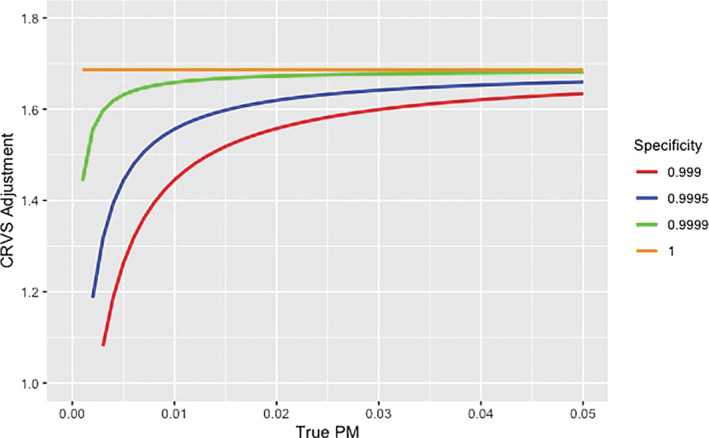
CRVS adjustment for different values of specificity, calculated at different levels of true PM when sensitivity is fixed at the global estimate of 0.593

Figure 4 shows the relationship between true PM and the estimated CRVS adjustment factors, for specific values of specificity to illustrate their effect on the CRVS adjustment factor. When specificity equals one, the CRVS adjustment factor equals one over sensitivity, hence lower sensitivity results in a higher adjustment; conversely higher sensitivity results in a lower adjustment. When specificity is less than one, while keeping sensitivity fixed, the adjustment factor decreases with decreasing true PM. This effect is due to an increasing share of false positive maternal deaths among all deaths, and a decreasing share of false negative deaths. In other words, as the true PM decreases, the number of non‐maternal deaths reported as maternal increases while the number of maternal deaths reported as non‐maternal decreases. This relationship implies that keeping specificity and sensitivity constant in extrapolations will result in changing adjustment factors as the true PM changes. Specifically, the adjustment factor will decrease if the true PM decreases in forward projections. Similarly, when using a fixed value of sensitivity and specificity, the adjustment factor associated with these values will depend on the value of the true PM.

Figure 4 also highlights that small differences in specificity, for a given value of sensitivity, result in notable differences in CRVS adjustment factor, especially at low PM. These differences are due to the large share of non‐maternal deaths, and thus the relatively larger share of false positives, among all deaths.

### 4.3 Country estimates

**FIGURE 5:**
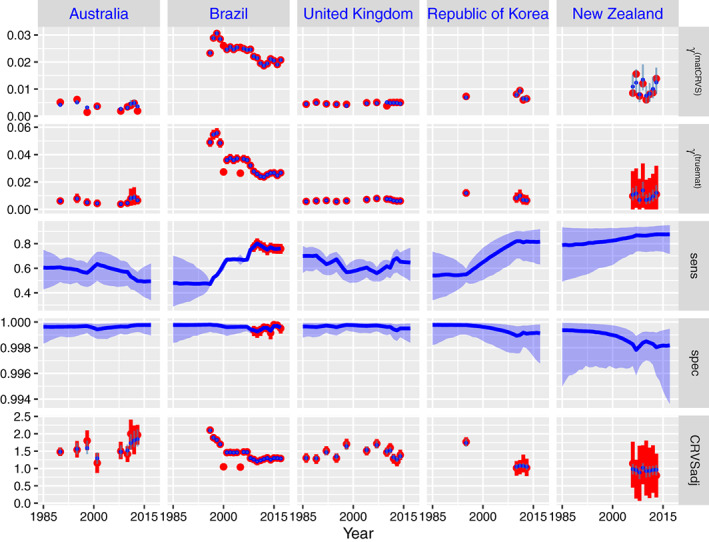
Illustration of BMis model data and estimates for Australia, Brazil, the United Kingdom, Republic of Korea, and New Zealand. Parameters plotted are CRVS‐based PM, true PM, sensitivity, specificity, and CRVS adjustment factors. The plots include: (1) Observed data with associated observation‐based 80% confidence intervals (red), (2) posterior estimates with 80% credible intervals (blue)

Sensitivity, specificity and CRVS adjustment estimates are shown for selected countries in Figure 5. Results for all countries are included in Appendix Section S6.5. In the plots, posterior estimates (blue) are shown with observed data (red) during the estimation period.

For the selected countries in Figure 5, given by Australia, Brazil, the United Kingdom, Republic of Korea, and New Zealand, estimates of sensitivity range from 0.468 in Brazil to 0.87 in New Zealand, and estimates of specificity range from 0.997 in New Zealand to 0.9998 in Australia. The results illustrates how uncertainty in estimates of sensitivity and specificity depends on (i) what information is available, (ii) the number of deaths in the country, and (iii) the observation years. In some countries, breakdown information on false positive and false negative deaths is available. An example country is Brazil, where sensitivity and specificity are recorded for recent years. Resulting estimates in those years are more certain than estimates in Brazil for earlier years (1996‐2008). In addition to availability of data, the number of deaths in the country also determines the uncertainty in estimated sensitivity and specificity. For example, data in New Zealand is very uncertain due to the small number of maternal deaths and total number of deaths to women of reproductive age. Lastly, uncertainty in sensitivity and specificity increases in years further away from years with data. For example in Brazil and New Zealand, uncertainty increases in backprojections prior to the earliest observation year. In Australia and the Republic of Korea, we see more uncertainty in long periods without data.

## 5 CONCLUSION

In this article, we presented a Bayesian hierarchical random walk model to assess maternal mortality misclassification errors in the CRVS with uncertainty. The model is based on the assessment of sensitivity and specificity of maternal mortality reporting, and captures differences therein between countries and within countries over time. Validation exercises suggest that the model performs well in terms of predicting CRVS‐based PM for country‐periods without specialized studies.

The new model improves upon limitations of the 2015 UN MMEIG approach. In the UN MMEIG 2015 round of estimation, for countries with specialized studies that overlapped with CRVS data, adjustments were calculated directly from available data (ie, the study's reported PM to CRVS‐based PM) and kept constant in extrapolations. The rationale for keeping adjustments constant in the 2015 approach for countries with studies was to implement “no change in quality of reporting.” However, when measuring quality of reporting in terms of sensitivity and specificity, the adjustment is not constant but varies with the true PM when keeping quality metrics constant, as illustrated in Figure 4. The BMis model‐based approach to obtaining adjustment factors improves upon this limitation of the UN MMEIG 2015 approach because its projections, which are based on constant sensitivity and specificity, are aligned with the assumption of constant quality of reporting. Model validation suggests that the new approach reduces errors in predicted CRVS‐based PM, in both validation exercises, that is, 20% random exclusions and leaving out the last observation. Finally, uncertainty assessments differ between the old and new approach. In the old approach, uncertainty in adjustments was assumed to be around 50% for all country‐periods. In the new approach, uncertainty in the adjustment factor follows from the uncertainty in the estimates for sensitivity and specificity and resulting adjustments are more certain in settings with recent information about quality of reporting. Validation exercises suggests that the new model is well calibrated.

Substantively, we estimate that sensitivity of maternal death reporting globally is 0.593 (0.523, 0.659)—in other words, that more than half the number of maternal deaths are reported as such—and that there are substantial differences in sensitivity and specificity across countries and within countries over time. More information is needed to understand why some CRVS systems have high or low estimates of sensitivity and specificity, to guide improvements in such systems for improved monitoring and to allow for more precise predictions of misclassification for country‐years without specialized studies.

Our work contributes to the statistics literature by proposing a bivariate hierarchical temporal model that captures differences in sensitivity and specificity across populations and over time and a likelihood function for data provided in aggregated form. The modeling approach allows for extrapolations to periods with missing data and accounts for temporal autocorrelation in the estimation of sensitivity and specificity. In this article, we applied the approach to the estimation of misclassification errors in maternal mortality in CRVS systems. Its application would extend naturally to the estimation of misclassification in other cause fractions where golden‐standard data are available for a subset of years. The approach can also be considered for other sets of populations or data systems, such as subnational estimation, where populations are defined by geographical regions within countries, and health information systems.

## Supporting information


**Appendix S1** Supplementary material

## Data Availability

The data that support the findings of this study are available upon request from the corresponding author and the World Health Organization.
