# Supplementary material for: Estimating misclassification errors in the reporting of maternal mortality in national civil registration vital statistics systems: A Bayesian hierarchical bivariate random walk model to estimate sensitivity and specificity for multiple countries and years with missing data
Source: Stat Med. 2022 Feb 14;41(14):2483–96. doi: 10.1002/sim.9335 (PMC9303473; doi:10.1002/sim.9335)
Supplement: Supplementary file 1 — Appendix S1 Supplementary material [file SIM-41-2483-s001.pdf]

6 Appendix

6.1 Definitions

Term	Description
Maternal death	The death of a woman whilst pregnant or within 42 days of termination of pregnancy, irrespective of the duration and site of the pregnancy, from any cause related to or aggravated by the pregnancy or its management but not from accidental or incidental causes define with the International Statistical Classification of Diseases and Related Health Problems 10th revision (ICD-10)
CRVS	Civil registration vital statistics, national death registration statistics
Specialized Study	A study conducted for the purpose of assessing the extent of misclassification within the CRVS and/or the extent of "missingness" of maternal deaths.
BMat	Bayesian maternal mortality estimation model, used by the UN MMEIG. BMat 2019 refers to the model used in the 2019 estimation round.
True positive maternal death	A maternal death correctly classified as maternal within CRVS.
True negative maternal death	A non-maternal death correctly classified as non-maternal within CRVS.
False positive maternal death	A non-maternal death misclassified as maternal within CRVS.
False negative maternal death	A maternal death misclassified as non-maternal within CRVS.
Sensitivity	(1) True positive rate, (2) Number of correctly classified maternal deaths over the true number of maternal deaths within CRVS systems.
Specificity	(1) True negative rate, (2) Number of correctly classified non-maternal deaths over the true number of non-maternal deaths within CRVS systems.
Missed/unregistered maternal death	A maternal death unregistered (missed) within CRVS, and therefore, unreported.
PM	The proportion of maternal deaths out of the total deaths to women of reproductive age (15-49).
CRVS-based PM	The proportion of CRVS-reported maternal deaths out of the total deaths to women of reproductive age within CRVS.
CRVS adjustment	Ratio of true PM to CRVS-based PM.

Table 4: Definitions

6.2 Compilation of specialized studies data

6.2.1 Summary of systematic review process

The objective of the review was to assess the level of misclassification reported by national official agencies for all WHO Member States. In other words, what is the level of incorrect reporting of maternal deaths in national official CRVS reporting, e.g. what is the difference between official reported number of maternal deaths versus the number of maternal deaths identified through special maternal mortality studies, confidential enquiries and surveillance systems etc. And to what extent is the incorrect reporting of maternal death due to misclassification versus missed or unregistered maternal deaths?

This review identified studies that fulfilled inclusion criteria as follows:

	**Inclusion Criteria**
Population	Women of reproductive age (15-49 years) who died during pregnancy or up to one year after termination of pregnancy, irrespective of duration and the site of the pregnancy, from any cause.
Concept	Assessment of misclassification of maternal deaths by CRVS systems.
Study design	Cross-sectional study and retrospective cohort
Context	All WHO Member States reporting CRVS data

In addition, the following criteria has to be met for inclusion:

1. study is nationally representative;
2. mid-years of reported data are after 2000;
3. there is a matched comparison with CRVS data available in the study or in the WHO Mortality Database.

6.2.1.1 Search Strategy The search strategy was conducted for all relevant existing literature based on search terms relevant to the research questions restricted to the years 1990-2016, using the following online bibliographic databases: PubMed/MEDLINE, EMBASE, Global Index Medicus, EBSCO, Web of Science and Popline. The searches were conducted without any language restrictions. Search terms are included in Box at the end of this document. A hand search was also conducted on all WHO Member States Ministries of Health (MoH) websites to identify pertinent MoH maternal mortality and confidential inquiries reports.

6.2.1.2 Data Extraction Data were extracted from full-text journal articles and reports which met the inclusion criteria. Data were extracted using a Microsoft Excel database. Information retrieved from the included studies included country, years assessed, study objectives, methodology /study design, number of maternal deaths, information on misclassification and incompleteness when available. Specifically, extraction focused on the assessment of the following:

1. The process by which the study retrieved and reviewed information on maternal deaths, including data source descriptions, definitions used by study, and whether the study reviewed all deaths to women of reproductive age or a description of the subset of deaths collected.
2. The number of maternal deaths, any information pertaining to misclassification of maternal cause of death by the CRVS system, any information regarding missed deaths by maternal cause.
3. Breakdown of maternal deaths by maternal cause of death was extracted if reported.

6.2.2 Compilation of data

The summary data compilation diagram in Figure 6 provides information on the number of study documents and associated study observations both identified and included by (1) systematic review, (2) WHO maternal mortality database, and (3) information obtained from follow-up surveys and country consultation. Lastly, it reports the number of studies excluded and reason for exclusion at each stage of the screening process. Studies were excluded in 3 subsequent steps. Firstly, studies were excluded if they reported information that could not be used, i.e. if no information on maternal death counts in the CRVS or associated envelopes could be obtained (non-usable data). Secondly, a study was excluded if it was not nationally representative. Lastly, a study was excluded if an alternate study with more up-to-date or detailed information for the same country-period was available. For a complete set of references of the included study documents see Box 1 at the end of Peterson et al. (2019).[11]

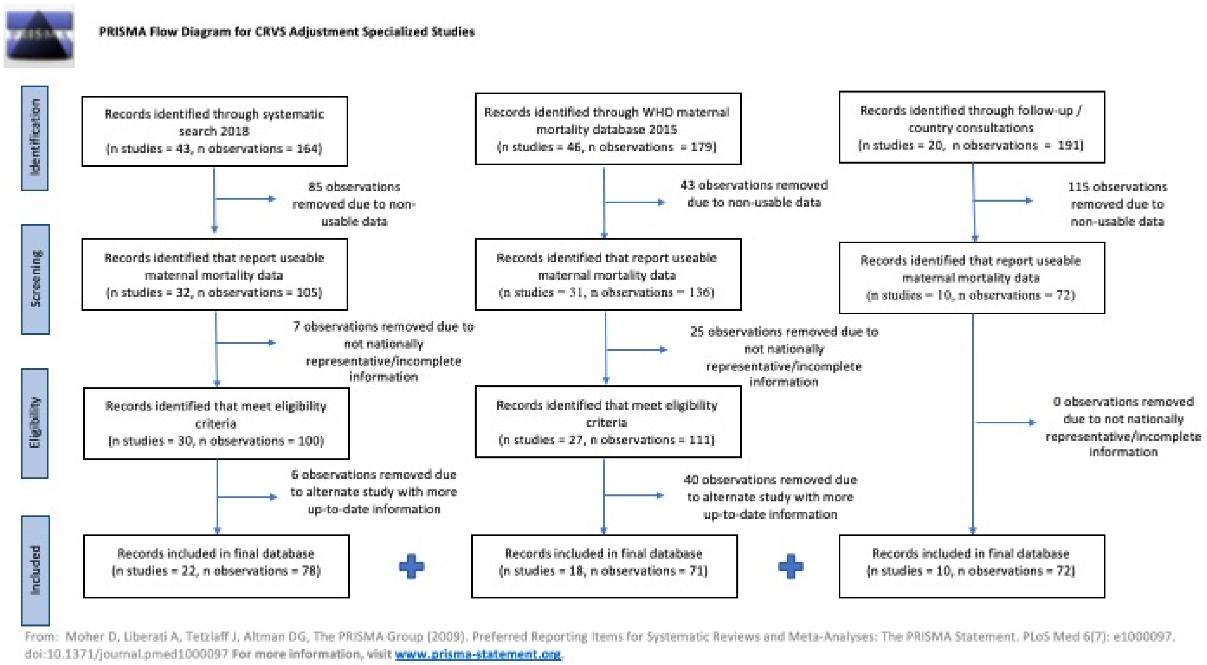

Figure 6: Summary of data compilation of specialized studies for inclusion in the BMis model. The numbers of studies mentioned refer to study documents.

6.3 Covariate plots

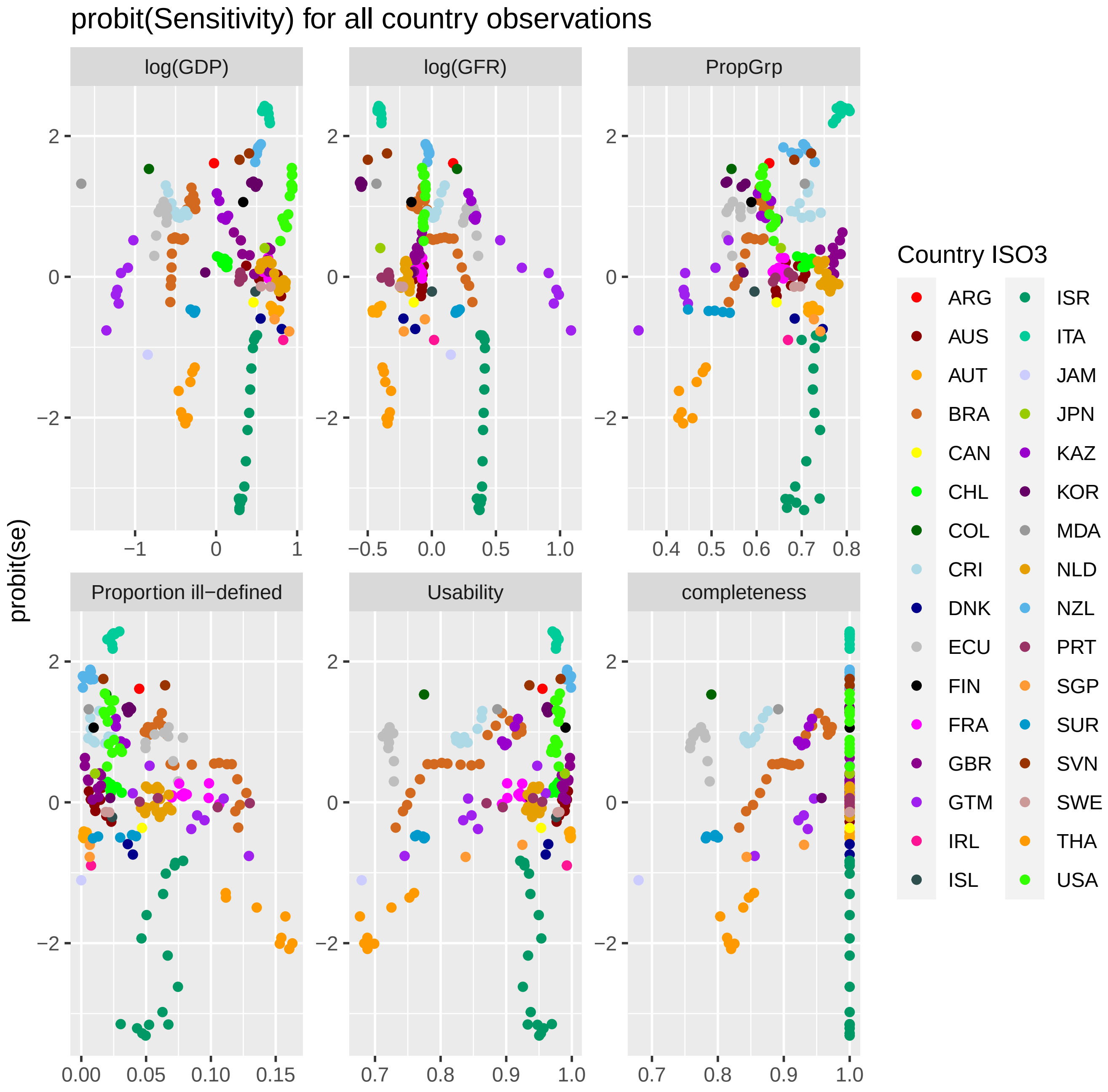

Figure 7: Estimates of sensitivity (on probit-scale) plotted against covariates.

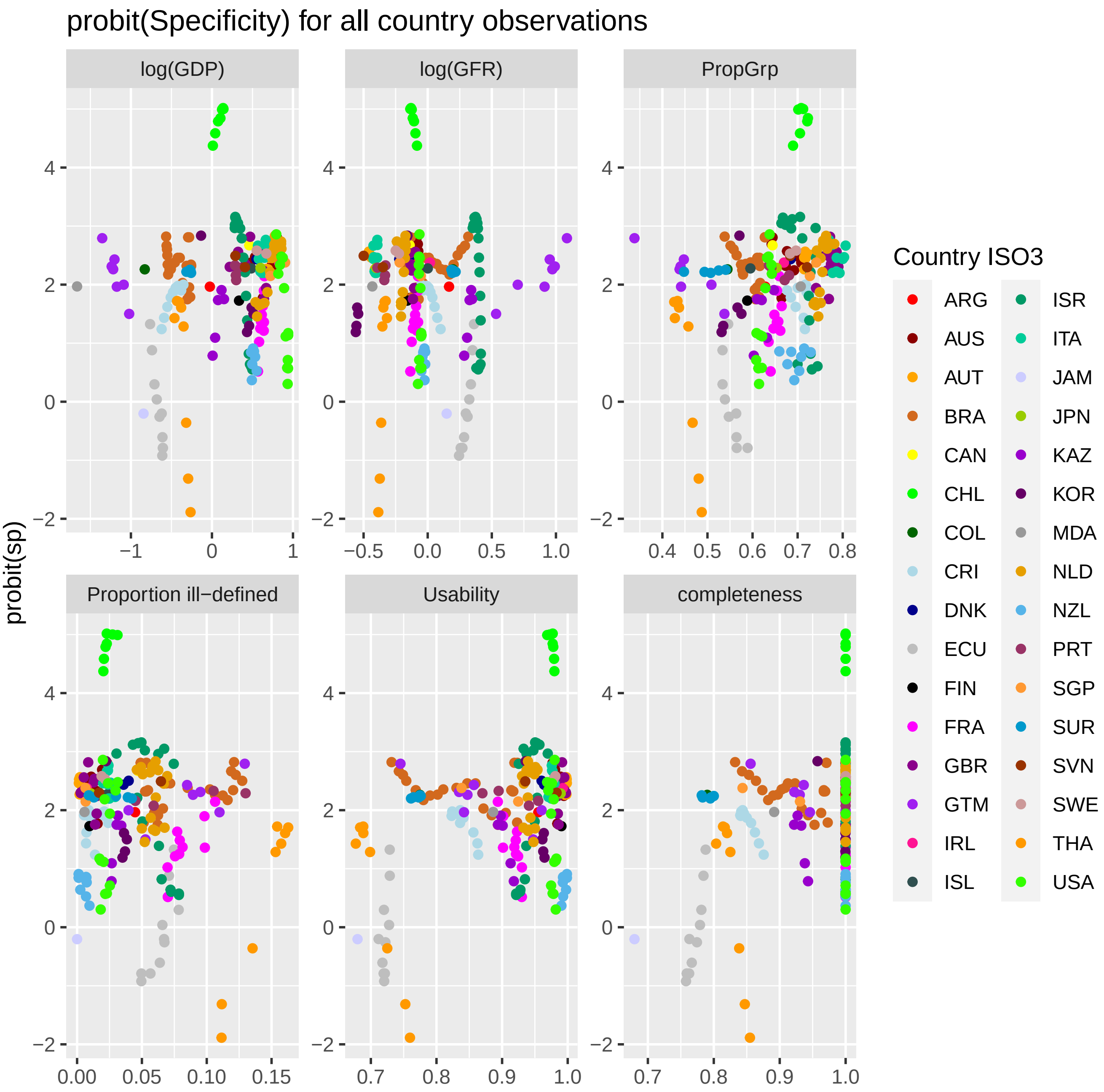

Figure 8: Estimates of specificity (on probit-scale) plotted against covariates.

6.4 Data generating processes and likelihood definitions

6.4.1 Likelihood function for non-overlapping counts

A simple illustration is shown in Table 5 for observation i, corresponding to country $c[i]$ and year $t[i]$, with reported non-overlapping multinomial death counts given by,

$z_i^{(T+)} = 272$	$z_i^{(F-)} = 2,762$	$z_i^{(\text{true})} = 3,034$
$z_i^{(F+)} = 1,171$	$z_i^{(T-)} = 57,821$	$z_i^{(\text{true-nonmat})} = 58,992$
$z_i^{(\text{mat})} = 1,443$	$z_i^{(\text{nonmat})} = 60,583$	$z_i^{(CRVS)} = 62,031$

Table 5: Example of non-overlapping counts reported for study i showing complete information on individual cell counts.

In the case of non-overlapping study counts $\boldsymbol{z}_i = \left(z_i^{(\text{T-})}, z_i^{(\text{T+})}, z_i^{(\text{F-})}, z_i^{(\text{F+})}\right)$, and the corresponding within CRVS probabilities $\boldsymbol{\gamma}_{c,t} = \left(\gamma_{c,t}^{(\text{T-})}, \gamma_{c,t}^{(\text{T+})}, \gamma_{c,t}^{(\text{F-})}, \gamma_{c,t}^{(\text{F+})}\right)$, we assume a multinomial density function given by:

$$p(\boldsymbol{z}_i | z_i^{(CRVS)}, \boldsymbol{\gamma}_{c[i],t[i]}) = \frac{z_i^{(CRVS)}!}{\prod_b z_i^{(b)}!} \prod_b \gamma_{c[i],t[i]}^{(b)\, z_i^{(b)}}. \tag{1}$$

with $z_i^{(\text{CRVS})} = \sum_b z_i^{(\text{b})}$.

6.4.2 Likelihood function for overlapping counts

Table 6 gives an illustration of a study with only reported information on the number of true maternal deaths within the CRVS, i.e., study-reported count of maternal deaths, $z_i^{(\text{true})} = z_i^{(\text{T+})} + z_i^{(\text{F-})}$, which overlaps with the CRVS-reported maternal deaths for the corresponding country-period, $z_i^{(\text{mat})} = z_i^{(\text{T+})} + z_i^{(\text{F+})}$. As such, we have reported overlapping marginal counts absent of information on individual cell counts.

$\tilde{z}_i^{(T+)} = ?$	$\tilde{z}_i^{(F-)} = ?$	$z_i^{(\text{true})} = 100$
$\tilde{z}_i^{(F+)} = ?$	$\tilde{z}_i^{(T-)} = ?$	$z_i^{(\text{true-nonmat})} = 900$
$z_i^{(\text{mat})} = 80$	$z_i^{(\text{nonmat})} = 920$	$z_i^{(tot)} = 1000$

Table 6: Example of overlapping counts reported for study i showing missing information on individual cell counts.

Let $\tilde{\boldsymbol{Z}}_i = \left(\tilde{Z}_i^{(\text{T-})}, \tilde{Z}_i^{(\text{T+})}, \tilde{Z}_i^{(\text{F-})}, \tilde{Z}_i^{(\text{F+})}\right)$ denote the unknown individual cell counts. Given the observed marginal counts, denoted $\boldsymbol{d}_i = (z_i^{(mat)}, z_i^{(true)})$, constraints are implied to individual cell counts such that they must satisfy the constraint that they sum to the marginal count, i.e.,

$$z_i^{(mat)} = \tilde{Z}_i^{(T+)} + \tilde{Z}_i^{(F+)}, \tag{2}$$

$$z_i^{(true)} = \tilde{Z}_i^{(T+)} + \tilde{Z}_i^{(F-)}. \tag{3}$$

Using direct sampling methods, we generate all combinations of 2 by 2 contigency tables of imputed multinomial cell counts, denoted $\tilde{\boldsymbol{z}}_i^{(s)}$ for $s = 1, \ldots, S[i]$, consistent with their respective marginal data and implied

constraints $\boldsymbol{d}_i$. Specifically, we define

$$\tilde{z}_i^{(mat)^{(s)}} = \tilde{z}_i^{(T+)^{(s)}} + \tilde{z}_i^{(F+)^{(s)}} = z_i^{(mat)}, \tag{4}$$

$$\tilde{z}_i^{(true)(s)} = \tilde{z}_i^{(T+)^{(s)}} + \tilde{z}_i^{(F-)^{(s)}} = z_i^{(true)}. \tag{5}$$

With the set of unique counts $\tilde{\boldsymbol{z}}_i^{(s)}$ for $s = 1, \ldots, S[i]$ for data $\boldsymbol{d}_i$, the likelihood function is obtained as follows:

$$p(\boldsymbol{d}_i|\boldsymbol{\gamma}_{c[i],t[i]}) = Pr(\boldsymbol{Z}_i = \tilde{\boldsymbol{z}}_i^{(1)} \cup \boldsymbol{Z}_i = \tilde{\boldsymbol{z}}_i^{(2)} \cup \ldots \cup \boldsymbol{Z}_i = \tilde{\boldsymbol{z}}_i^{(S[i])}|\boldsymbol{\gamma}_{c[i],t[i]}), \tag{6}$$

$$= \sum_{s=1}^{S[i]} Pr(\boldsymbol{Z}_i = \tilde{\boldsymbol{z}}_i^{(s)}|\boldsymbol{\gamma}_{c[i],t[i]}), \tag{7}$$

$$= \sum_{s=1}^{S[i]} p(\tilde{\boldsymbol{z}}_i^{(s)}|\boldsymbol{\gamma}_{c[i],t[i]}), \tag{8}$$

with the density for $Pr(\boldsymbol{Z}_i = \tilde{\boldsymbol{z}}_i^{(s)}|\boldsymbol{\gamma}_{c[i],t[i]}) = p(\tilde{\boldsymbol{z}}_i^{(s)}|\boldsymbol{\gamma}_{c[i],t[i]})$ as given in Eq. 1.

6.4.2.1 Exclusion of combinations with negligible probability The number of multinomial combinations consistent with $\boldsymbol{d}_i$ becomes large for a subset of studies. To improve computational efficiency in model fitting, we excluded combinations of $\tilde{\boldsymbol{z}}_i^{(s)}$ that have negligible probabilities of being the true combination underlying the aggregate counts. The likelihood is modified as follows:

$$p(\boldsymbol{d}_i|z_i^{(CRVS)}, \boldsymbol{\gamma}_{c[i],t[i]}) = \sum_{s=1}^{S[i]} p(\tilde{\boldsymbol{z}}_i^{(s)}|z_i^{(CRVS)}, \boldsymbol{\gamma}_{c[i],t[i]}) \cdot k_i^{(s)}, \tag{9}$$

where $k_i^{(s)} = 0$ for combination $\tilde{\boldsymbol{z}}_i^{(s)}$ with negligible probability of being the true combination underlying the aggregate counts, $k_i^{(s)} = 1$ otherwise.

We use $k_i^{(s)}$ to exclude combinations that are associated with values of specificity below 0.97. This cut-off was based on an assessment of available data. Combined across country-years with data, we found that the proportion of country-years of specificity below 0.99 is negligible, less than 0.1%, and the proportion with specificity below 0.97 is 0. Hence, based on available data, the lower bound on specificity of 0.97 excludes combinations with negligible probabilities of being true.

To account for the stochastic uncertainty associated with the imputed study counts, the exclusion indicator $k_i^{(s)}$ is defined using a tail percentile of the sampling distribution for the true negative counts $\tilde{Z}_i^{(\text{T-})}$. This sampling distribution is given by

$$\tilde{Z}_i^{(\text{T-})}|\lambda_i^{(-)}, z_i^{(\text{true})}, z_i^{(\text{CRVS})} \sim Binom\left(z_i^{(\text{CRVS})} - z_i^{(\text{true})}, \lambda_i^{(-)}\right),$$

and we define $q_i^{\lambda_i^{(-)}} = Bin_{2.5\%}\left(z_i^{(\text{CRVS})} - z_i^{(\text{true})}, \lambda_i^{(-)}\right)$ to be the 2.5th percentile of this distribution with sensitivity $\lambda_i^{(-)}$. Assuming that specificity $\lambda_i^{(-)} >> 0.97$, it follows that

$$q_i^{0.97} << q_i^{\lambda_i^{(-)}}.$$

Hence we find:

$$\text{Prob}\left(\tilde{Z}_i^{(\text{T-})} \leq q_i^{0.97}\right) << \text{Prob}\left(\tilde{Z}_i^{(\text{T-})} \leq q_i^{\lambda_i^{(-)}}\right) = 0.025.$$

We exclude combinations with negligible probability defined by $\tilde{z}_i^{(\text{T-})^{(s)}} \leq q_i^{(0.97)}$ by setting

$$k_i^{(s)} = 1\left(\tilde{z}_i^{(\text{T-})^{(s)}} > q_i^{(0.97)}\right),$$

such that $k_i^{(s)} = 0$ when $\tilde{z}_i^{(\text{T-})^{(s)}} \leq q_i^{(0.97)}$ and $k_i^{(s)} = 1$ otherwise.

6.5 Country plots

The plots include: 1. observed data with associated observation-based 80% confidence intervals (red), 2. posterior estimates with 80% credible intervals (blue).

Parameter Notation

- $\gamma^{(F-)}$: Probability of a false negative maternal death reported in CRVS.
- $\gamma^{(F+)}$: Probability of a false positive maternal death reported in CRVS.
- $\gamma^{(T-)}$: Probability of a true negative maternal death reported in CRVS.
- $\gamma^{(T+)}$: Probability of a true positive maternal death reported in CRVS.
- $\gamma^{(matCRVS)}$: Probability of a maternal death reported in CRVS.
- $\gamma^{(truemat)}$: Probability of a true maternal death reported in CRVS.
- $\gamma^{(FP/matvr)}$: Probability of a false positive death out of maternal deaths reported in CRVS.
- $\gamma^{(FN/truematvr)}$: Probability of a false negative death out of true maternal deaths reported in CRVS.
- $sens$: Probability of correctly identifying true maternal death reported in CRVS.
- $spec$: Probability of correctly identifying true non-maternal death reported in CRVS.
- $CRVSadj$: CRVS adjustment factor.

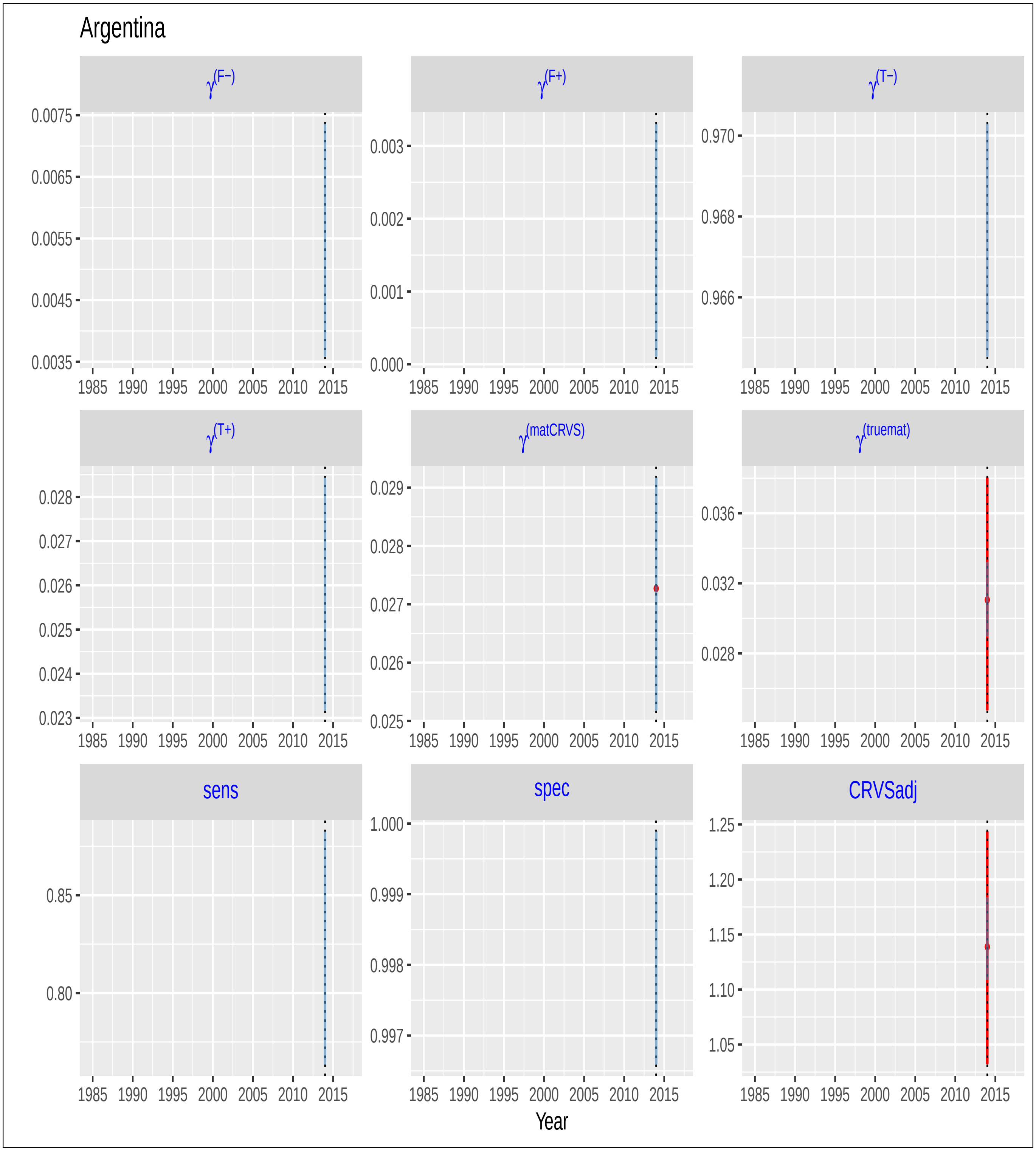

Argentina
γ(F−)
γ(F+)
γ(T−)
γ(T+)
γ(matCRVS)
γ(truemat)
sens
spec
CRVSadj
Year

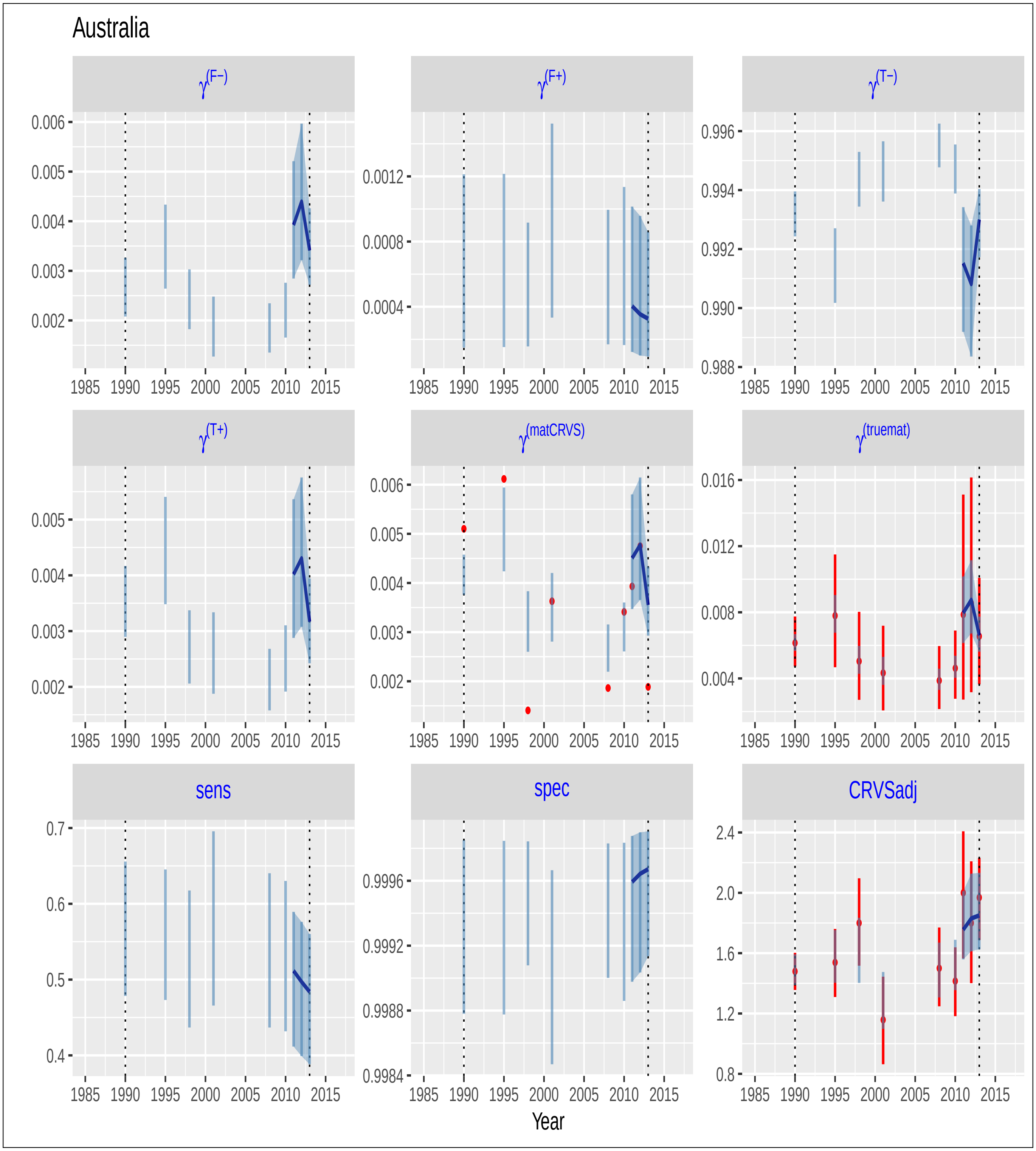

Australia
γ(F−)
γ(F+)
γ(T−)
γ(T+)
γ(matCRVS)
γ(truemat)
sens
spec
CRVSadj
1985 1990 1995 2000 2005 2010 2015
Year

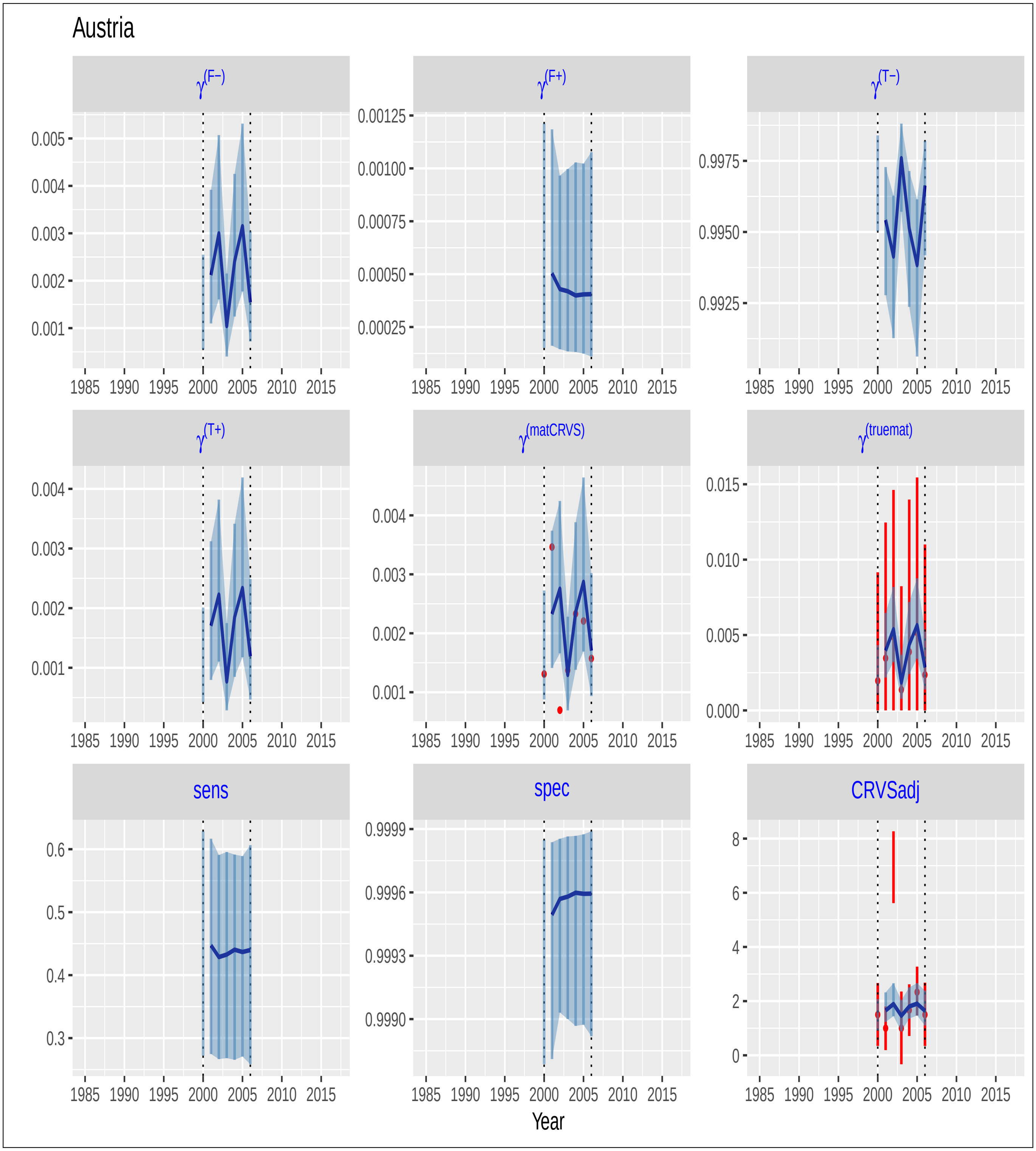

Austria
$\gamma^{(F-)}$
$\gamma^{(F+)}$
$\gamma^{(T-)}$
$\gamma^{(T+)}$
$\gamma^{(matCRVS)}$
$\gamma^{(truemat)}$
sens
spec
CRVSadj
Year

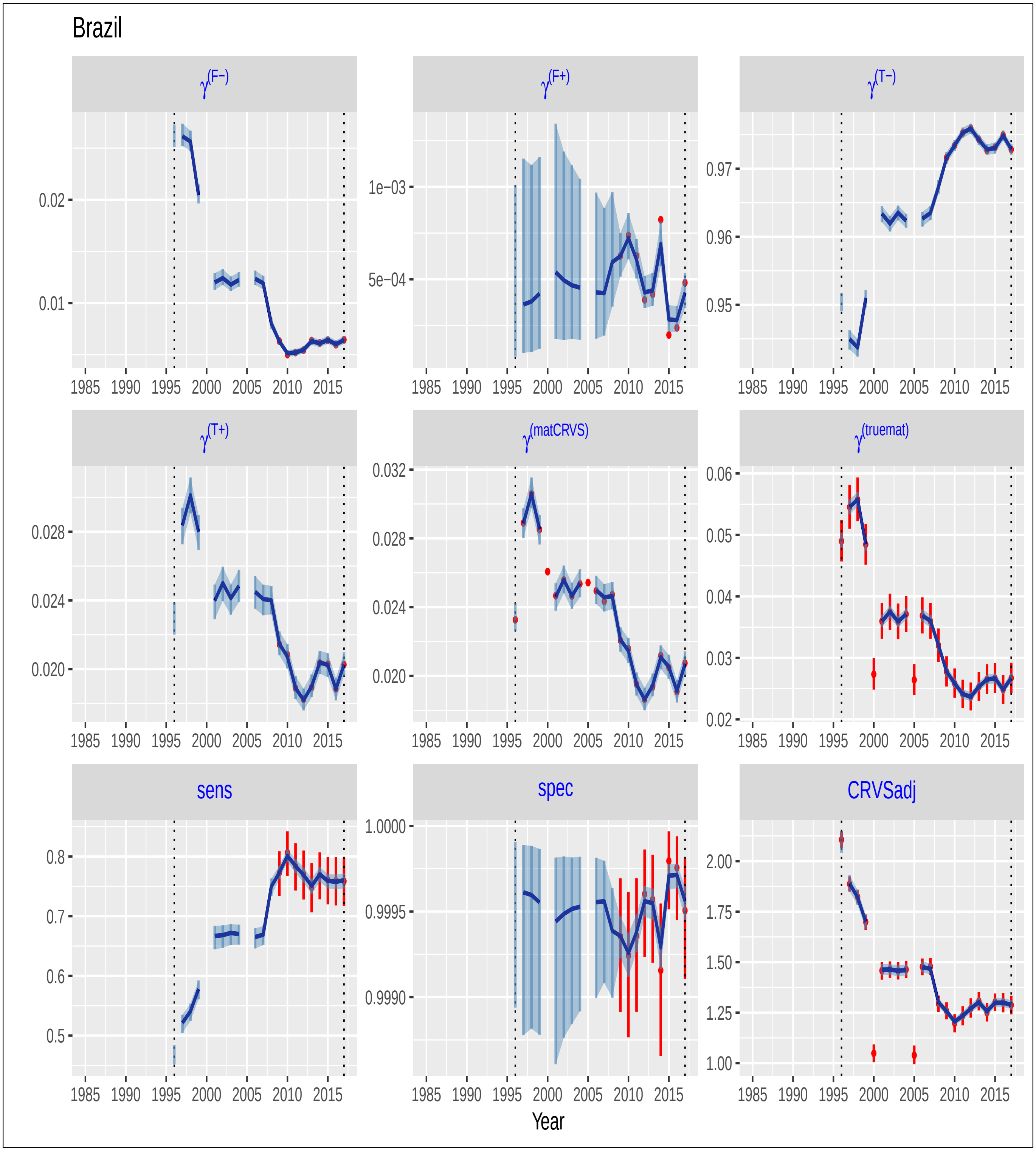
Brazil
γ(F−)
γ(F+)
γ(T−)
γ(T+)
γ(matCRVS)
γ(truemat)
sens
spec
CRVSadj
Year

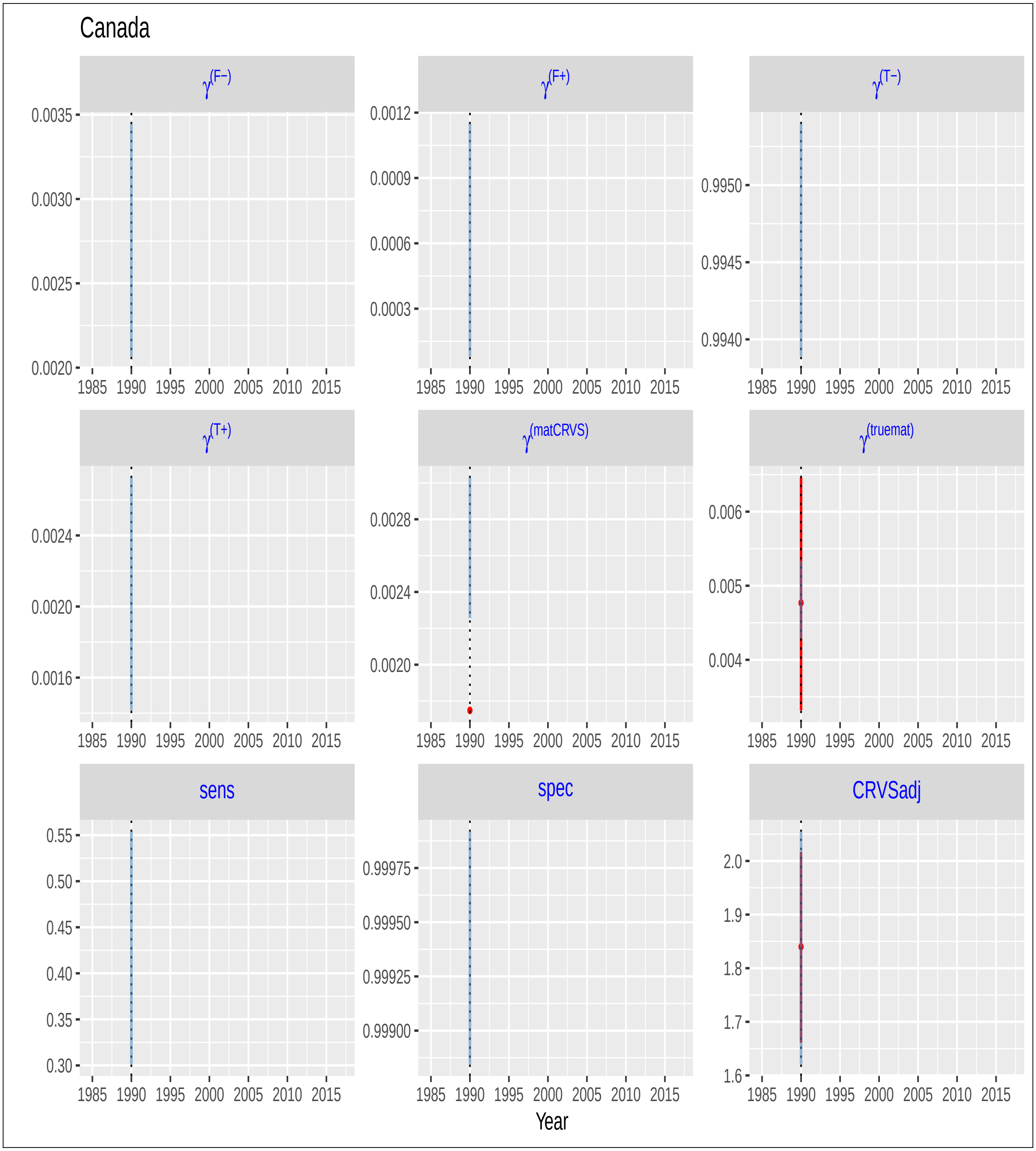

Canada
γ(F−)
γ(F+)
γ(T−)
γ(T+)
γ(matCRVS)
γ(truemat)
sens
spec
CRVSadj
0.0035
0.0030
0.0025
0.0020
0.0012
0.0009
0.0006
0.0003
0.9950
0.9945
0.9940
0.0024
0.0020
0.0016
0.0028
0.0024
0.0020
0.006
0.005
0.004
0.55
0.50
0.45
0.40
0.35
0.30
0.99975
0.99950
0.99925
0.99900
2.0
1.9
1.8
1.7
1.6
1985 1990 1995 2000 2005 2010 2015
Year

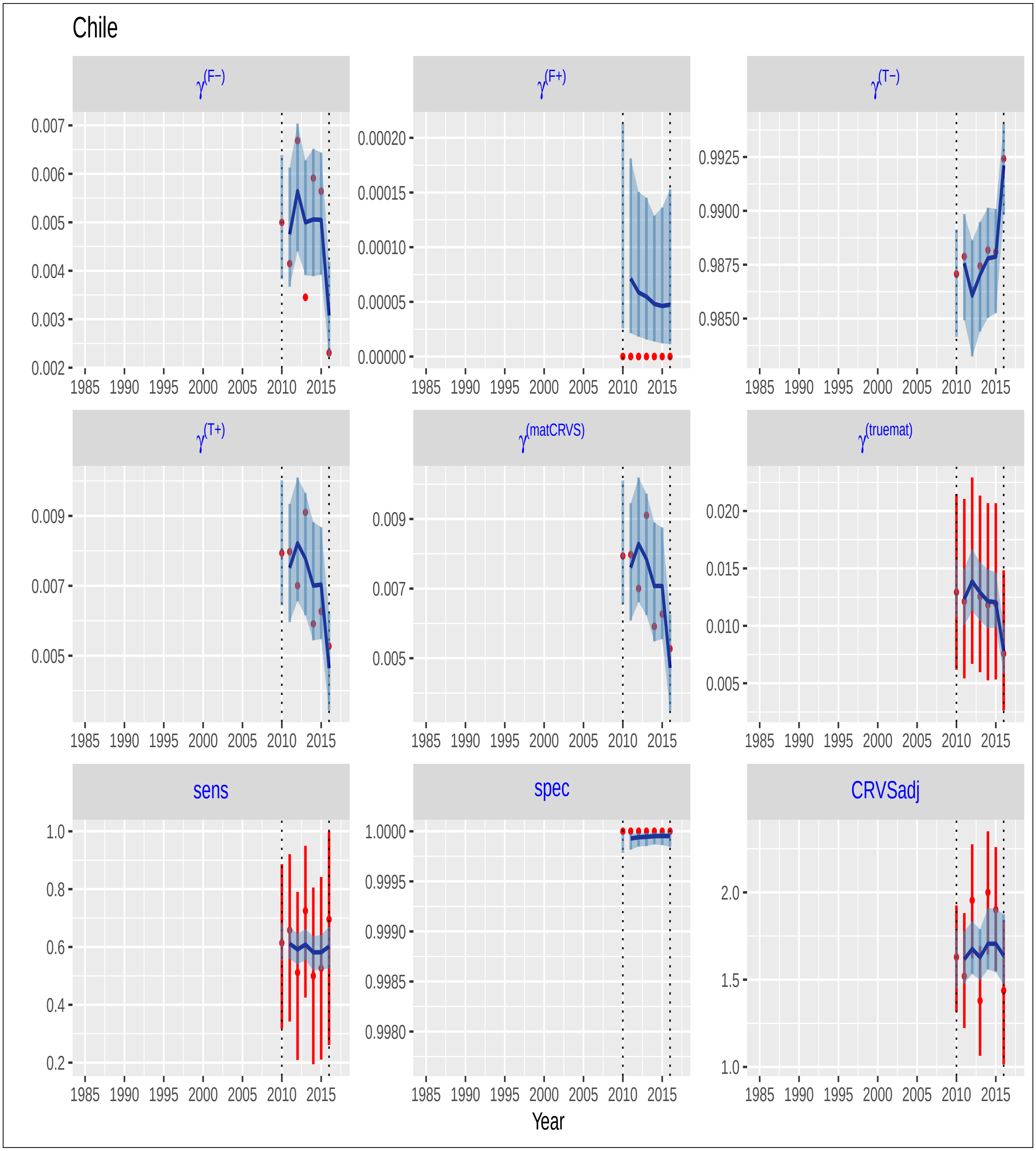
Chile
γ(F−)
γ(F+)
γ(T−)
γ(T+)
γ(matCRVS)
γ(truemat)
sens
spec
CRVSadj
Year

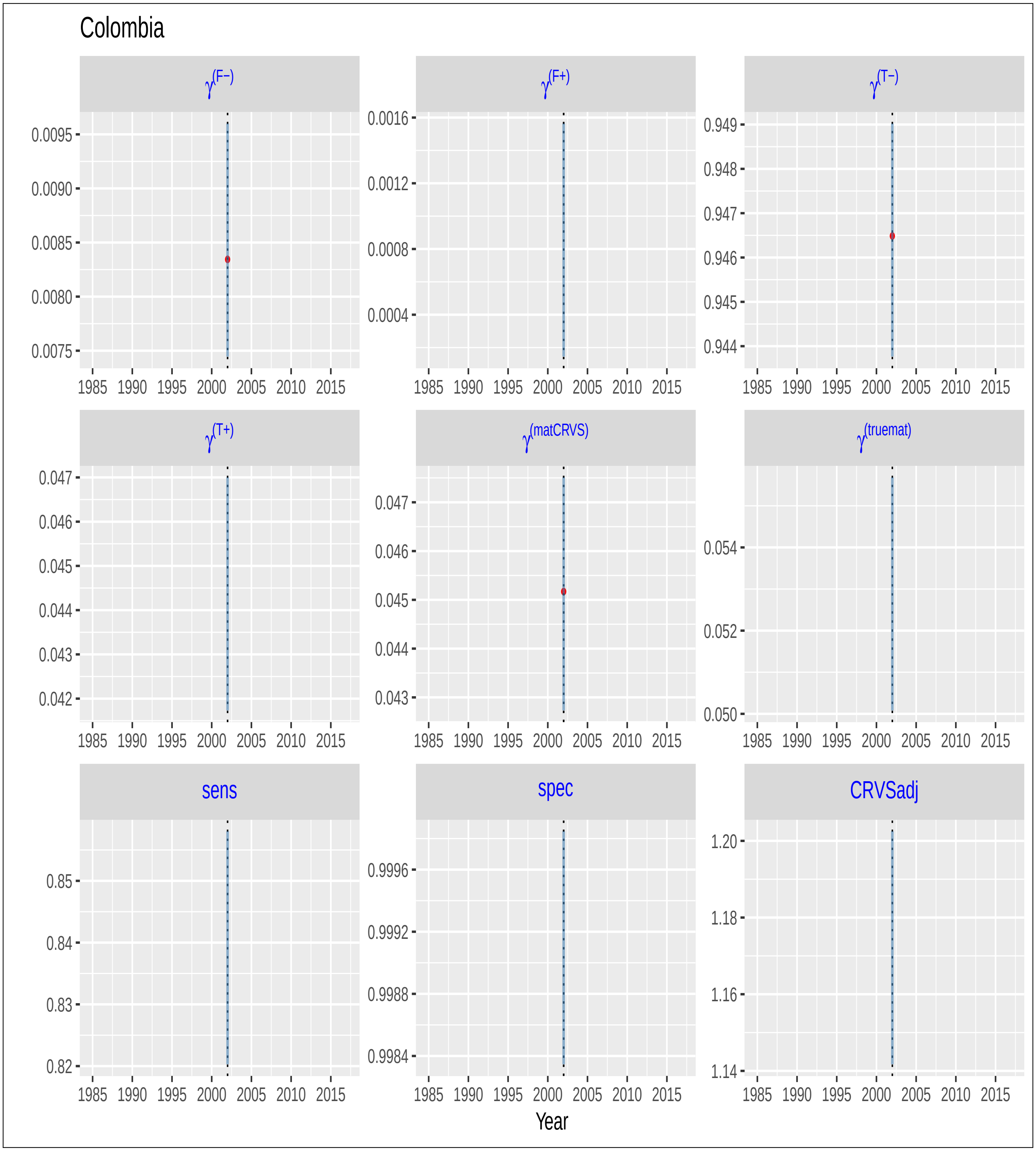

Colombia
γ(F−)
γ(F+)
γ(T−)
γ(T+)
γ(matCRVS)
γ(truemat)
sens
spec
CRVSadj
Year

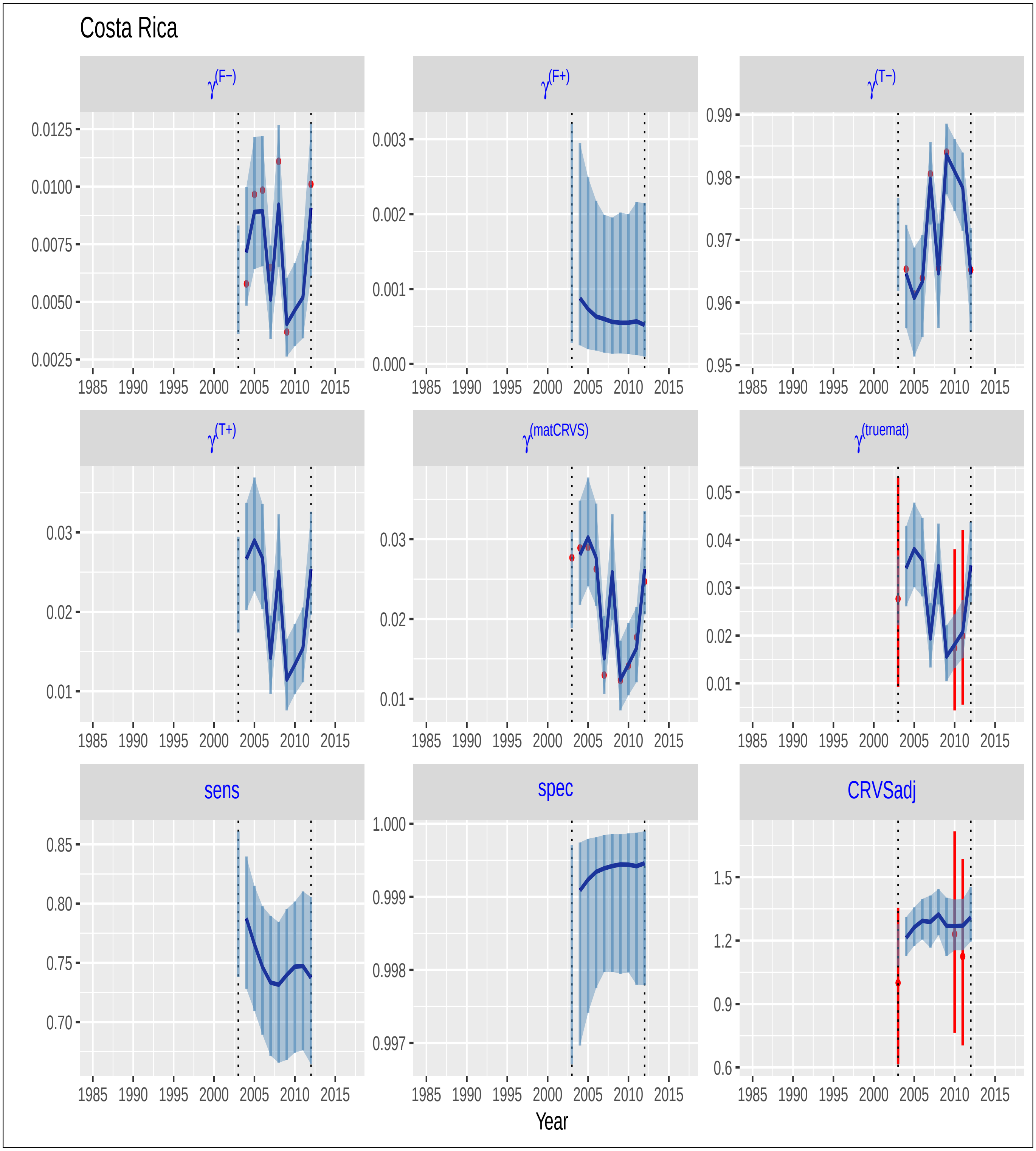

Costa Rica
$\gamma^{(F-)}$
$\gamma^{(F+)}$
$\gamma^{(T-)}$
$\gamma^{(T+)}$
$\gamma^{(matCRVS)}$
$\gamma^{(truemat)}$
sens
spec
CRVSadj
Year

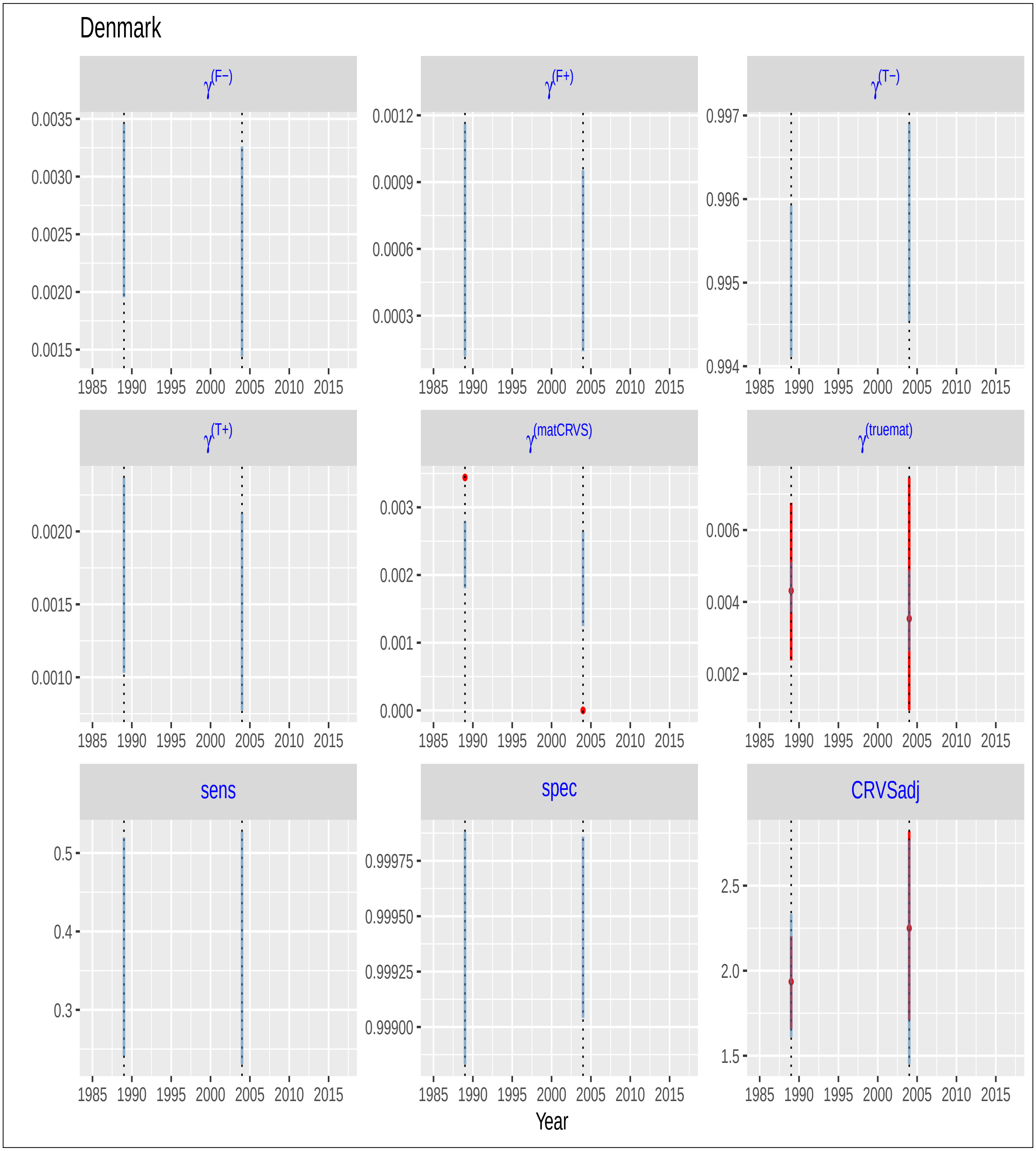

Denmark
γ(F−)
γ(F+)
γ(T−)
γ(T+)
γ(matCRVS)
γ(truemat)
sens
spec
CRVSadj
Year

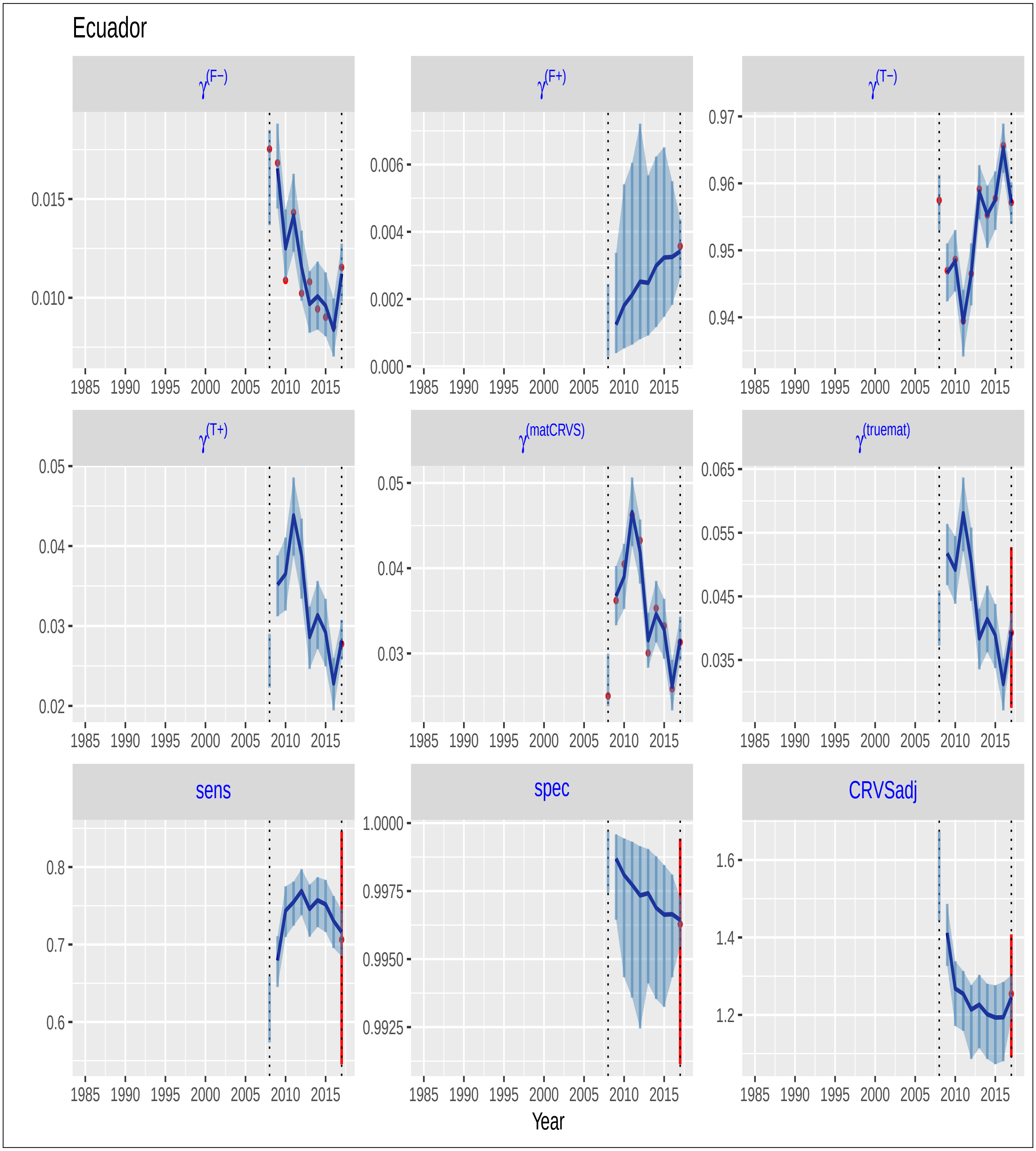

Ecuador
$\gamma^{(F-)}$
$\gamma^{(F+)}$
$\gamma^{(T-)}$
$\gamma^{(T+)}$
$\gamma^{(matCRVS)}$
$\gamma^{(truemat)}$
sens
spec
CRVSadj
Year

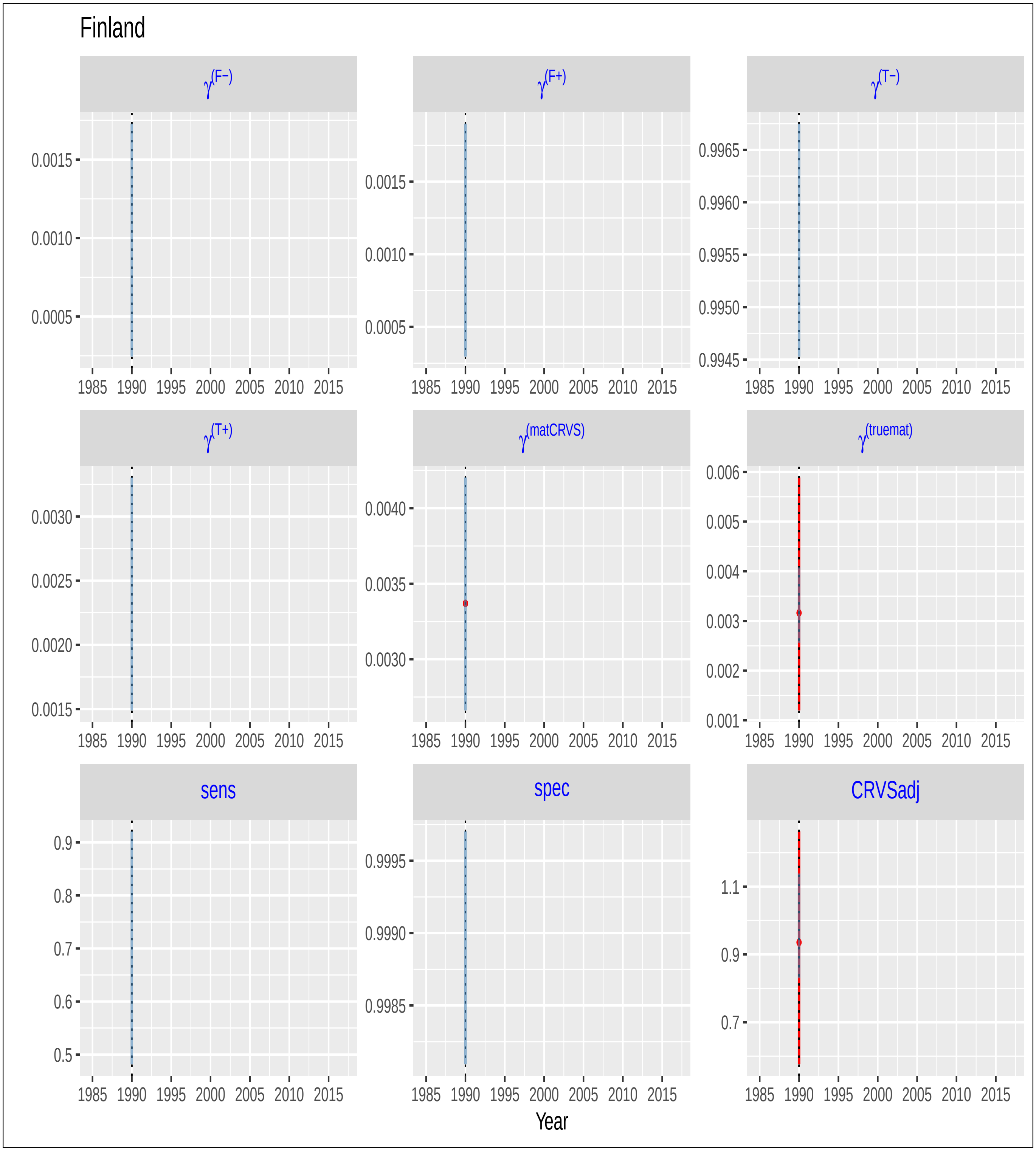

Finland
γ(F−)
γ(F+)
γ(T−)
γ(T+)
γ(matCRVS)
γ(truemat)
sens
spec
CRVSadj
Year
1985 1990 1995 2000 2005 2010 2015

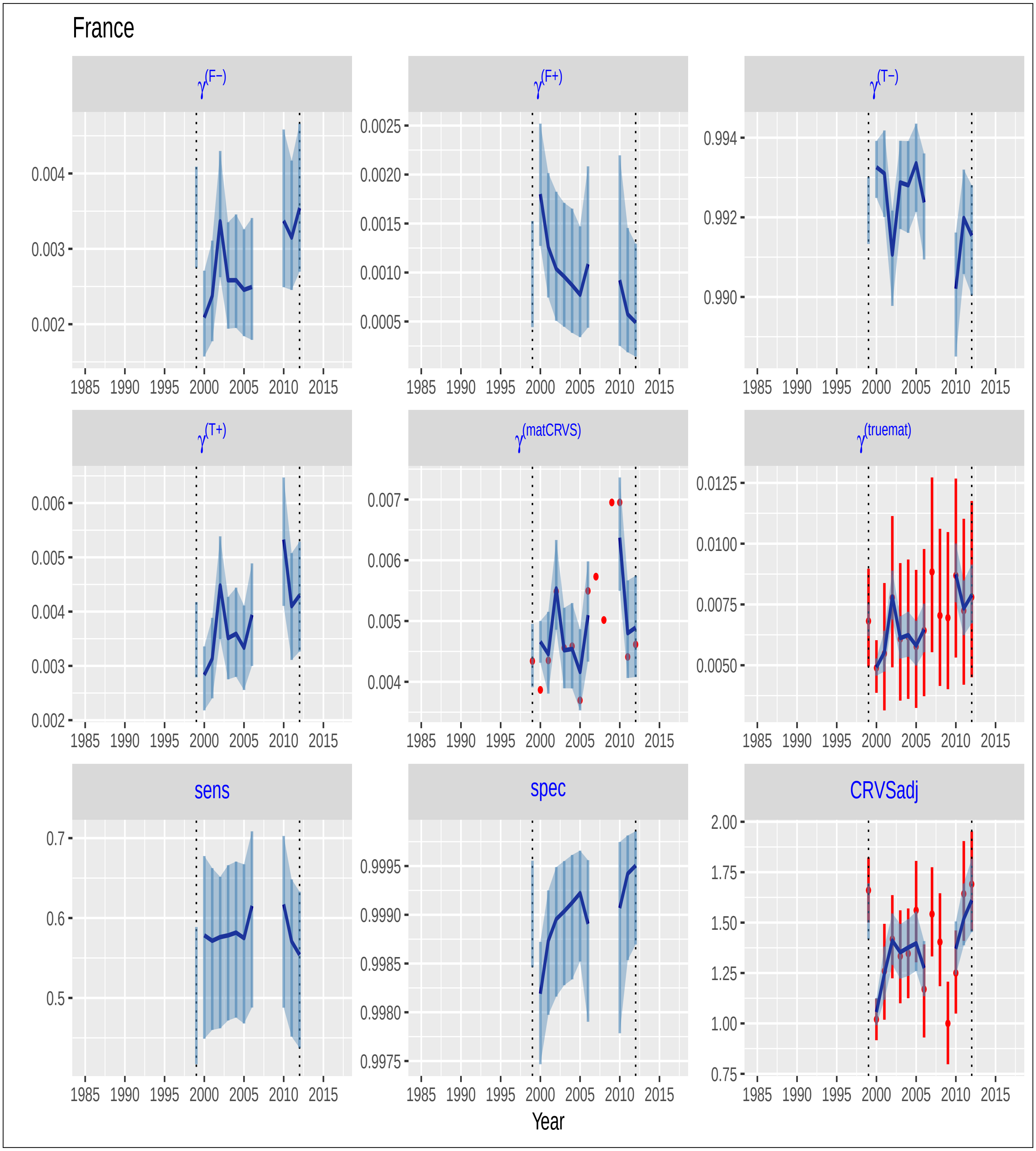

France
γ^(F−)
γ^(F+)
γ^(T−)
γ^(T+)
γ^(matCRVS)
γ^(truemat)
sens
spec
CRVSadj
Year
1985 1990 1995 2000 2005 2010 2015

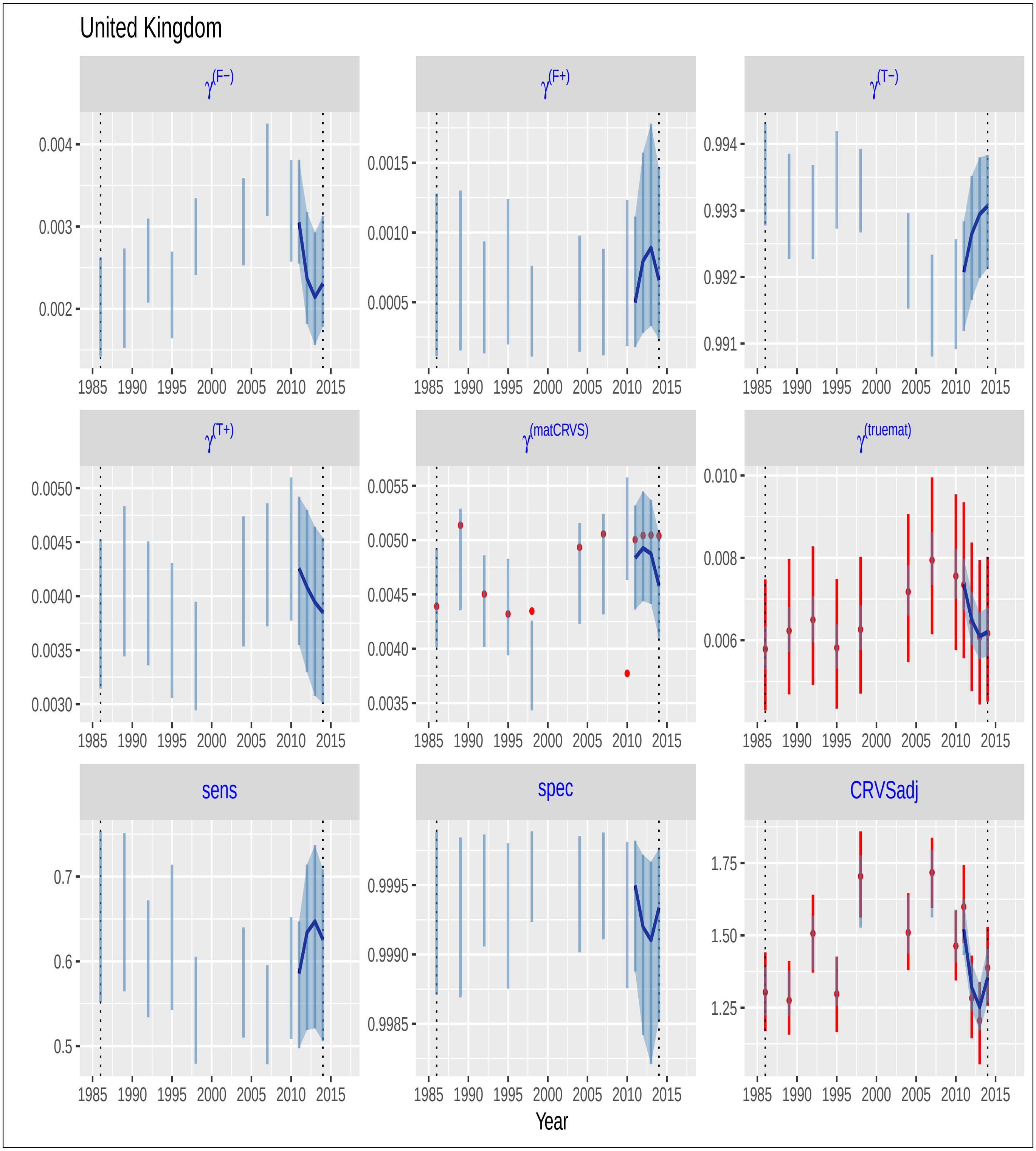

United Kingdom
$\gamma^{(F-)}$
$\gamma^{(F+)}$
$\gamma^{(T-)}$
$\gamma^{(T+)}$
$\gamma^{(matCRVS)}$
$\gamma^{(truemat)}$
sens
spec
CRVSadj
Year

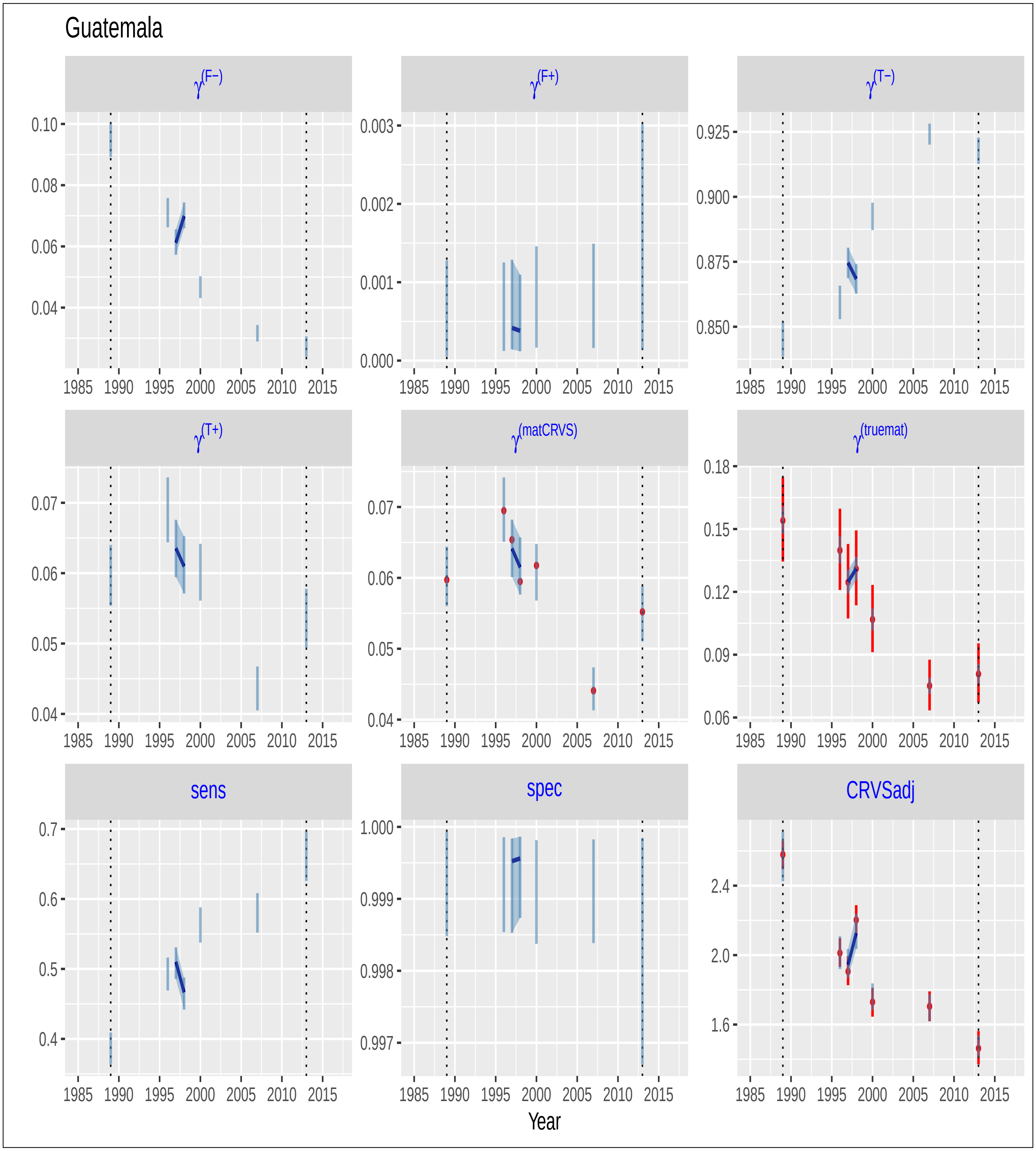

Guatemala
γ(F−)
γ(F+)
γ(T−)
γ(T+)
γ(matCRVS)
γ(truemat)
sens
spec
CRVSadj
Year

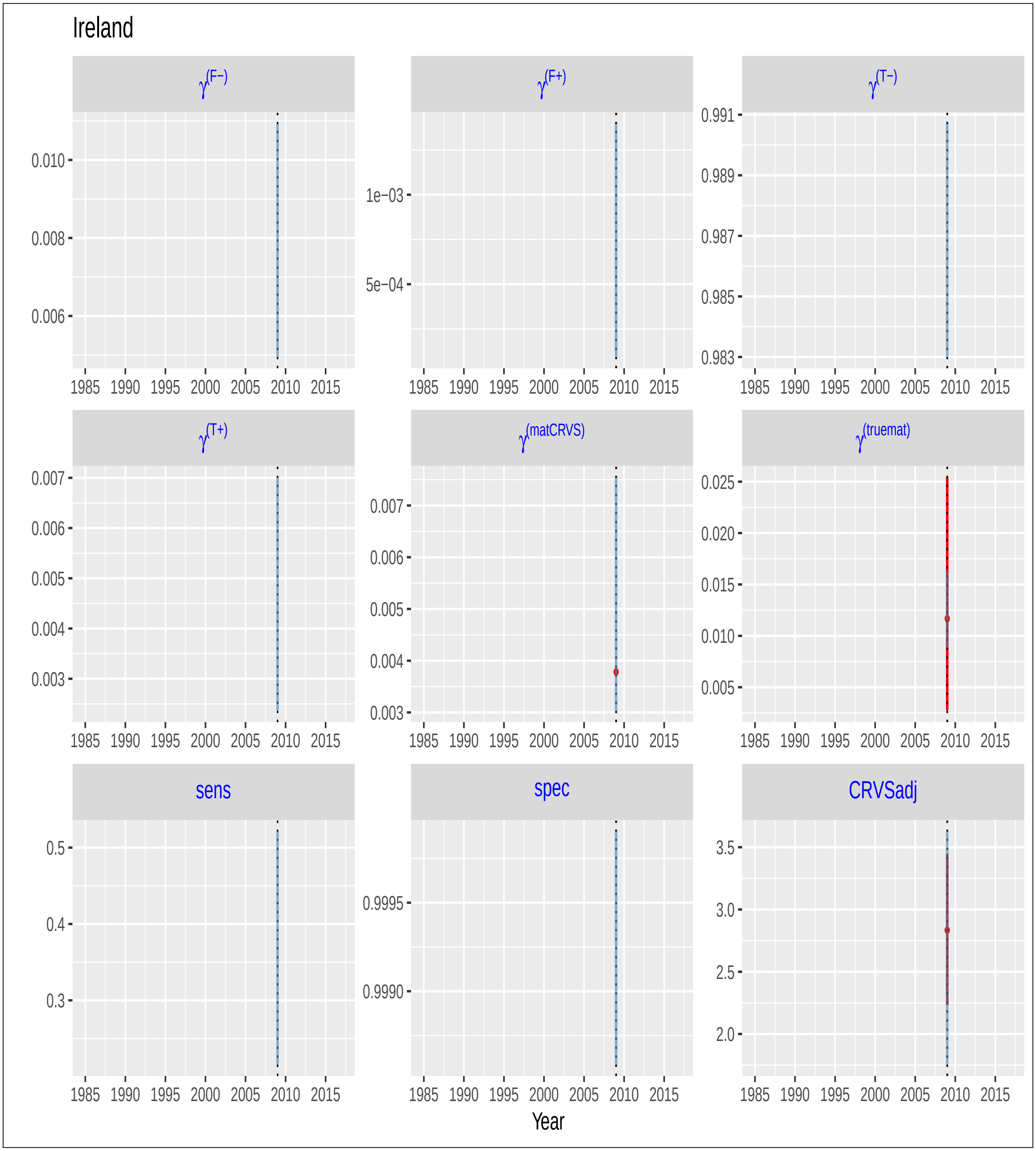

Ireland
$\gamma^{(F-)}$
$\gamma^{(F+)}$
$\gamma^{(T-)}$
$\gamma^{(T+)}$
$\gamma^{(matCRVS)}$
$\gamma^{(truemat)}$
sens
spec
CRVSadj
0.010
0.008
0.006
1e−03
5e−04
0.991
0.989
0.987
0.985
0.983
0.007
0.006
0.005
0.004
0.003
0.025
0.020
0.015
0.010
0.005
0.5
0.4
0.3
0.9995
0.9990
3.5
3.0
2.5
2.0
1985 1990 1995 2000 2005 2010 2015
Year

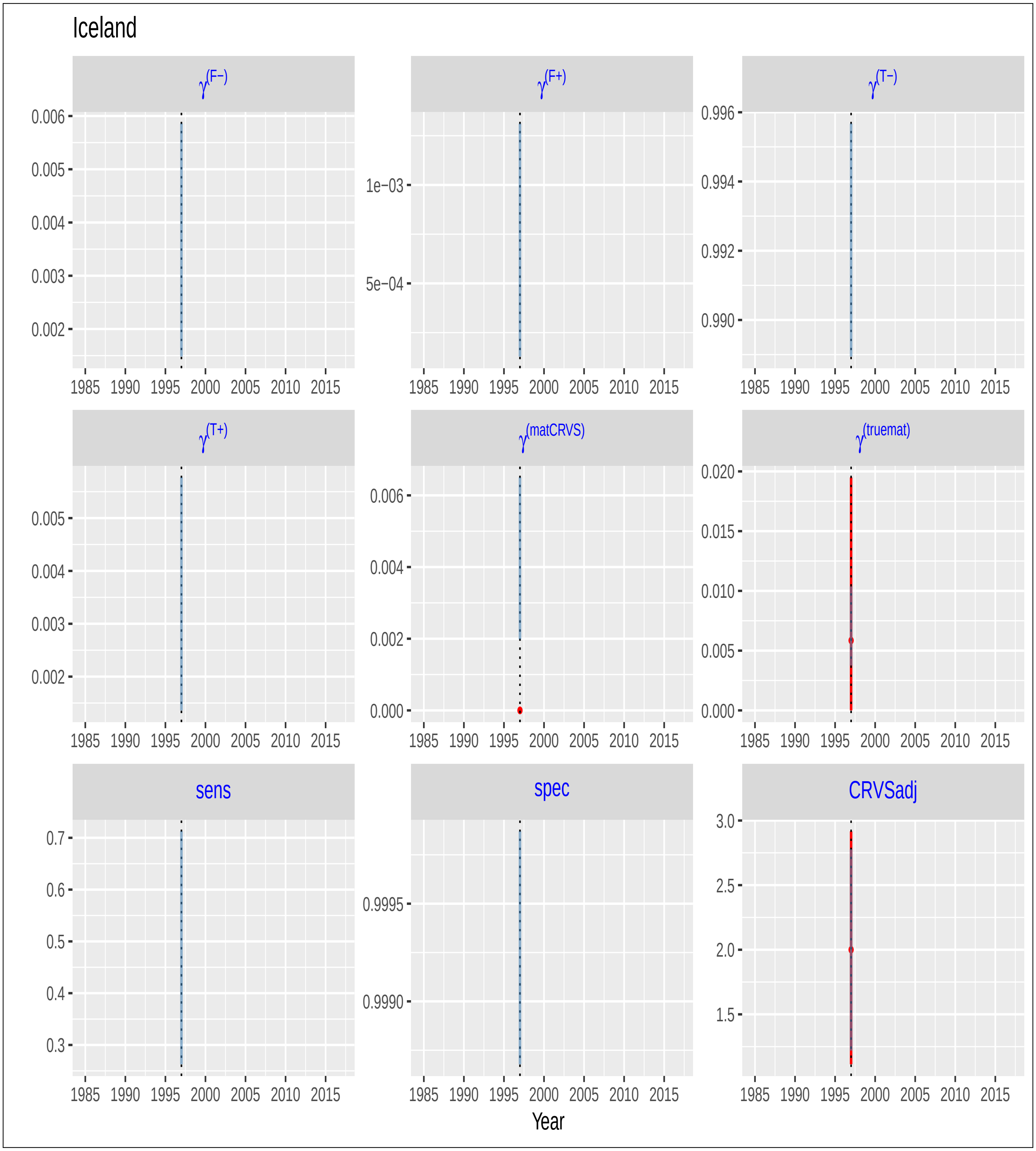

Iceland
γ(F−)
γ(F+)
γ(T−)
γ(T+)
γ(matCRVS)
γ(truemat)
sens
spec
CRVSadj
Year

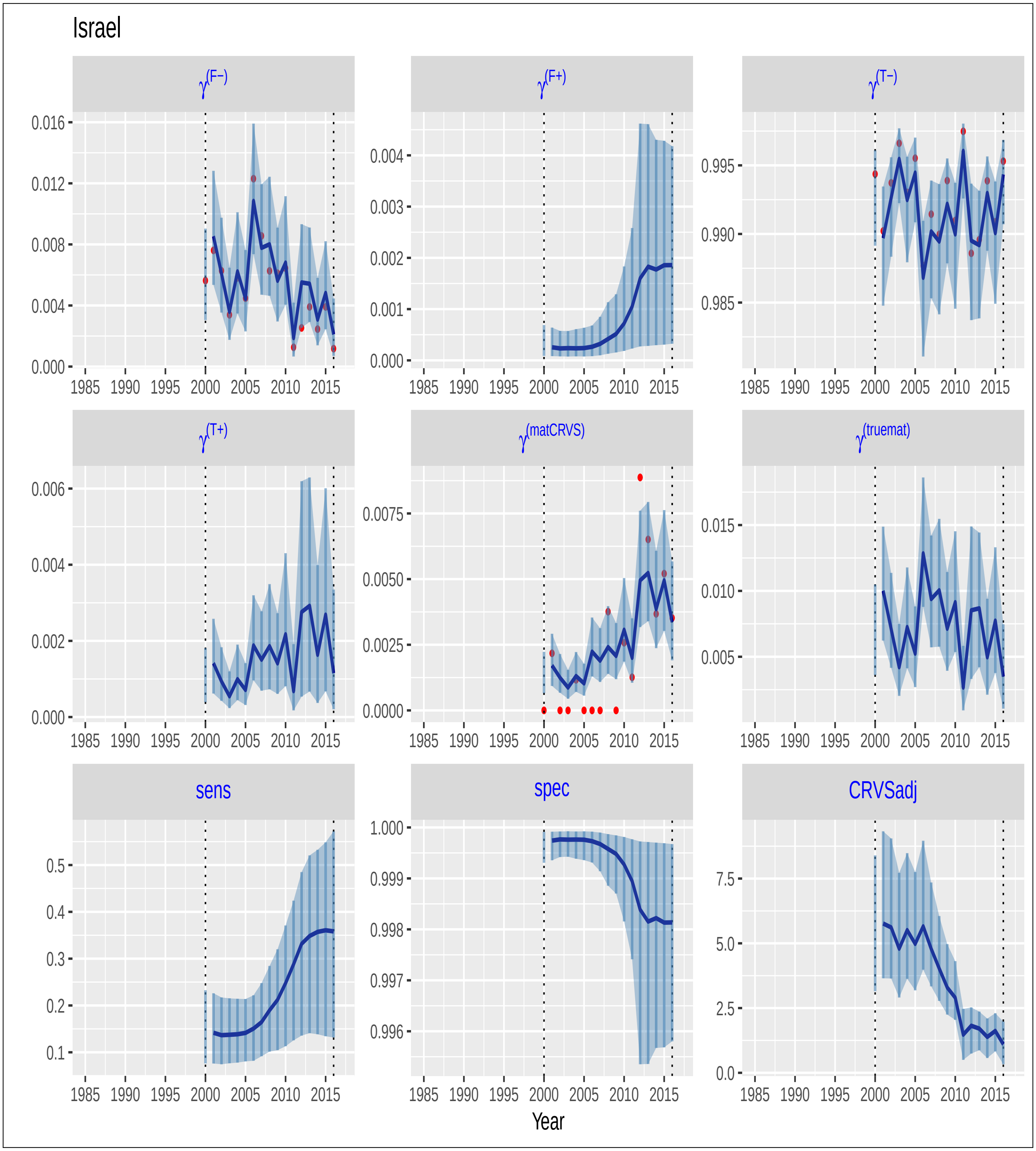
Israel
γ(F−)
γ(F+)
γ(T−)
γ(T+)
γ(matCRVS)
γ(truemat)
sens
spec
CRVSadj
0.016
0.012
0.008
0.004
0.000
0.004
0.003
0.002
0.001
0.000
0.995
0.990
0.985
0.006
0.004
0.002
0.000
0.0075
0.0050
0.0025
0.0000
0.015
0.010
0.005
0.5
0.4
0.3
0.2
0.1
1.000
0.999
0.998
0.997
0.996
7.5
5.0
2.5
0.0
1985 1990 1995 2000 2005 2010 2015
Year

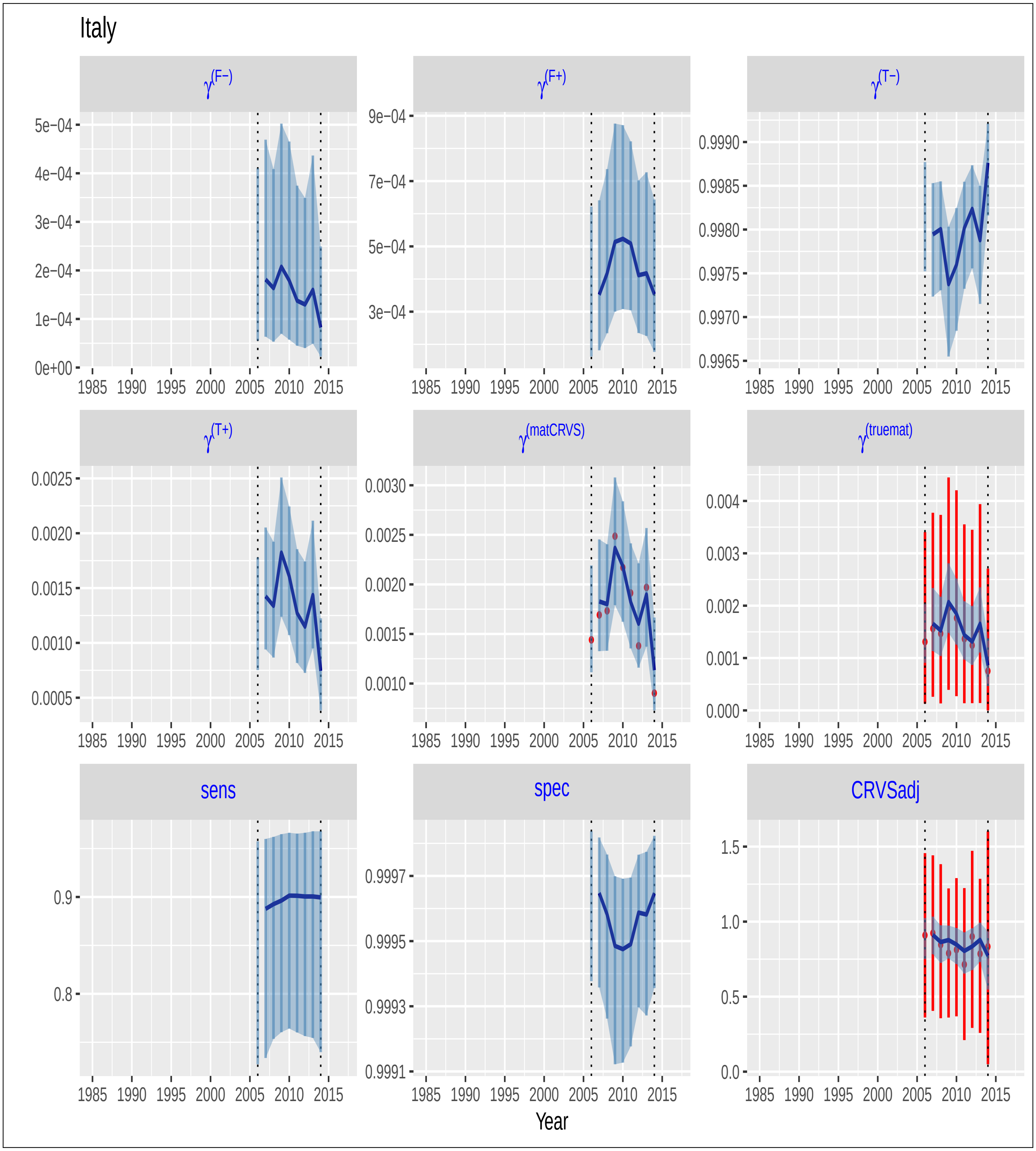
Italy
γ(F−)
γ(F+)
γ(T−)
γ(T+)
γ(matCRVS)
γ(truemat)
sens
spec
CRVSadj
Year

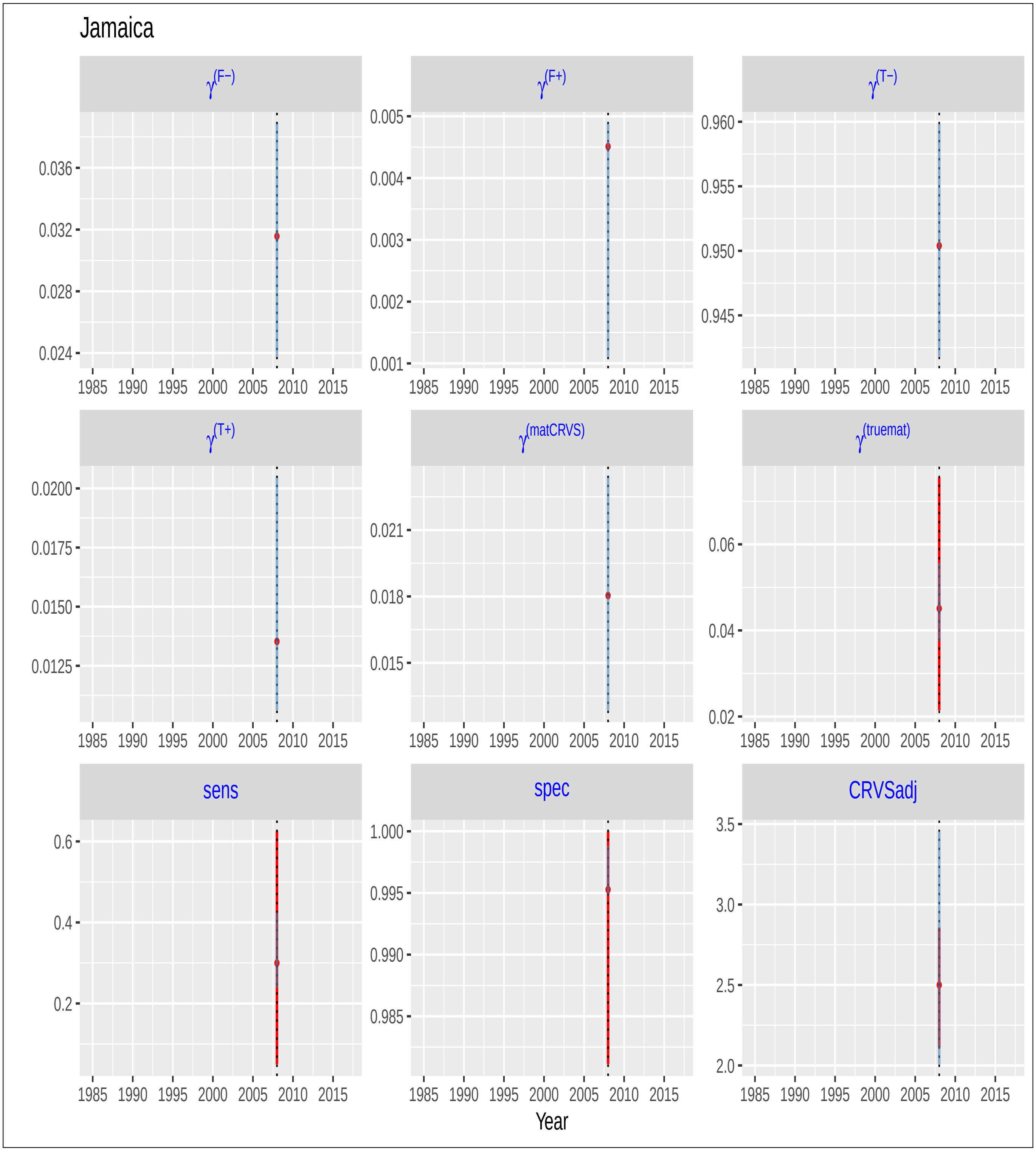

Jamaica
γ(F−)
γ(F+)
γ(T−)
γ(T+)
γ(matCRVS)
γ(truemat)
sens
spec
CRVSadj
Year

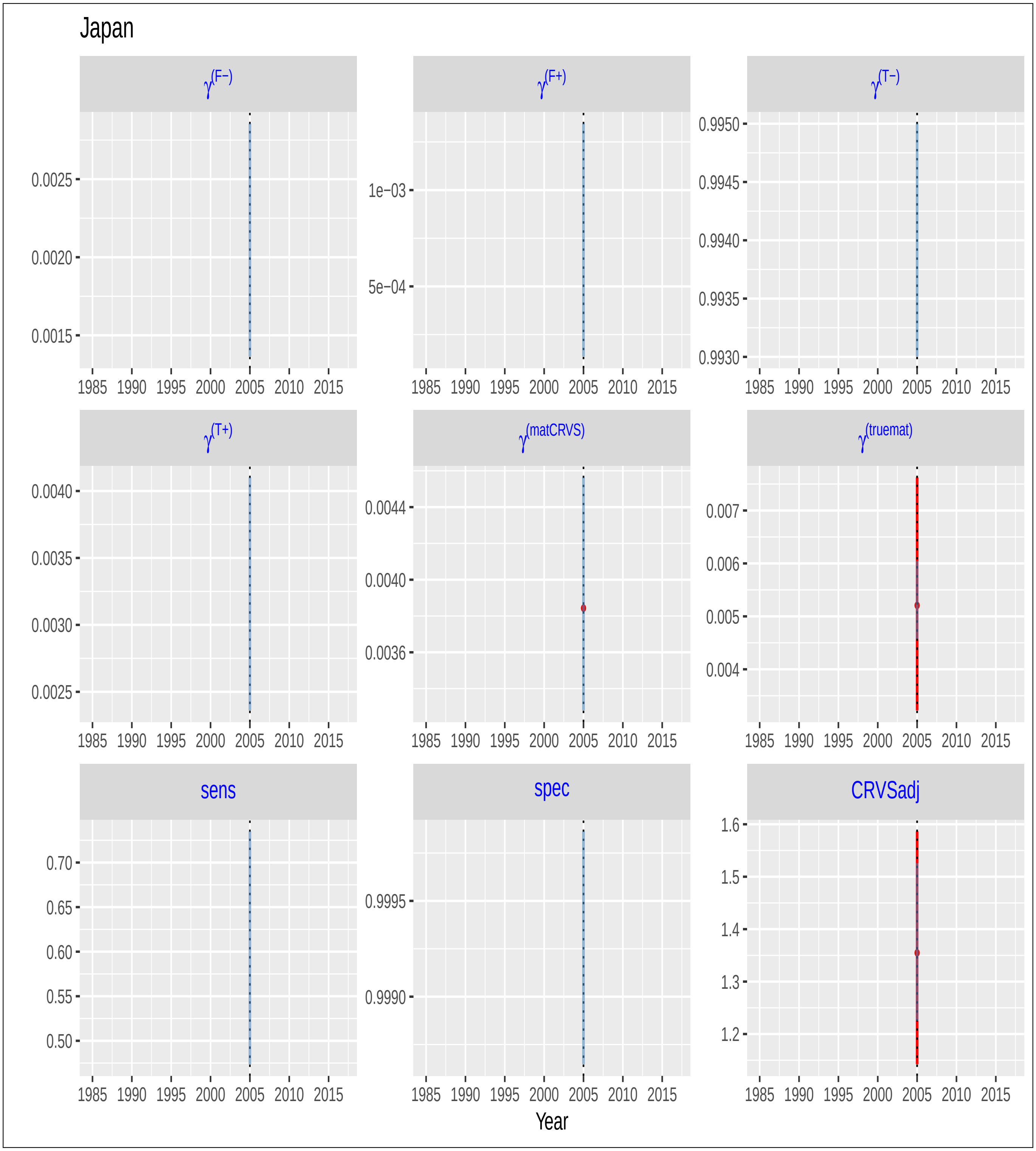

Japan
γ(F−)
0.0025
0.0020
0.0015
γ(F+)
1e−03
5e−04
γ(T−)
0.9950
0.9945
0.9940
0.9935
0.9930
γ(T+)
0.0040
0.0035
0.0030
0.0025
γ(matCRVS)
0.0044
0.0040
0.0036
γ(truemat)
0.007
0.006
0.005
0.004
sens
0.70
0.65
0.60
0.55
0.50
spec
0.9995
0.9990
CRVSadj
1.6
1.5
1.4
1.3
1.2
1985 1990 1995 2000 2005 2010 2015
Year

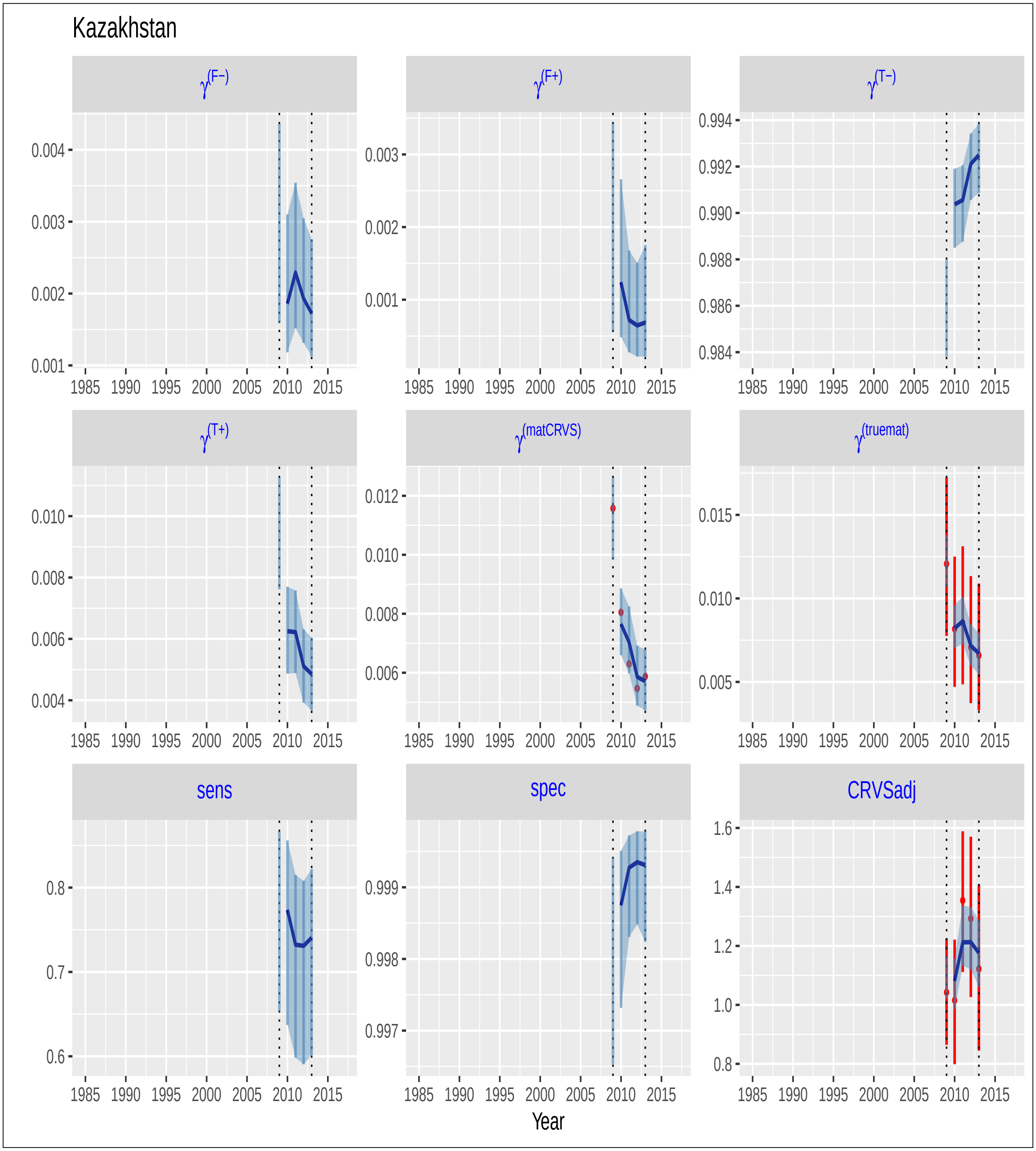

Kazakhstan
γ(F−)
γ(F+)
γ(T−)
γ(T+)
γ(matCRVS)
γ(truemat)
sens
spec
CRVSadj
0.004
0.003
0.002
0.001
0.994
0.992
0.990
0.988
0.986
0.984
0.010
0.008
0.006
0.012
0.015
0.005
0.8
0.7
0.6
0.999
0.998
0.997
1.6
1.4
1.2
1.0
1985 1990 1995 2000 2005 2010 2015
Year

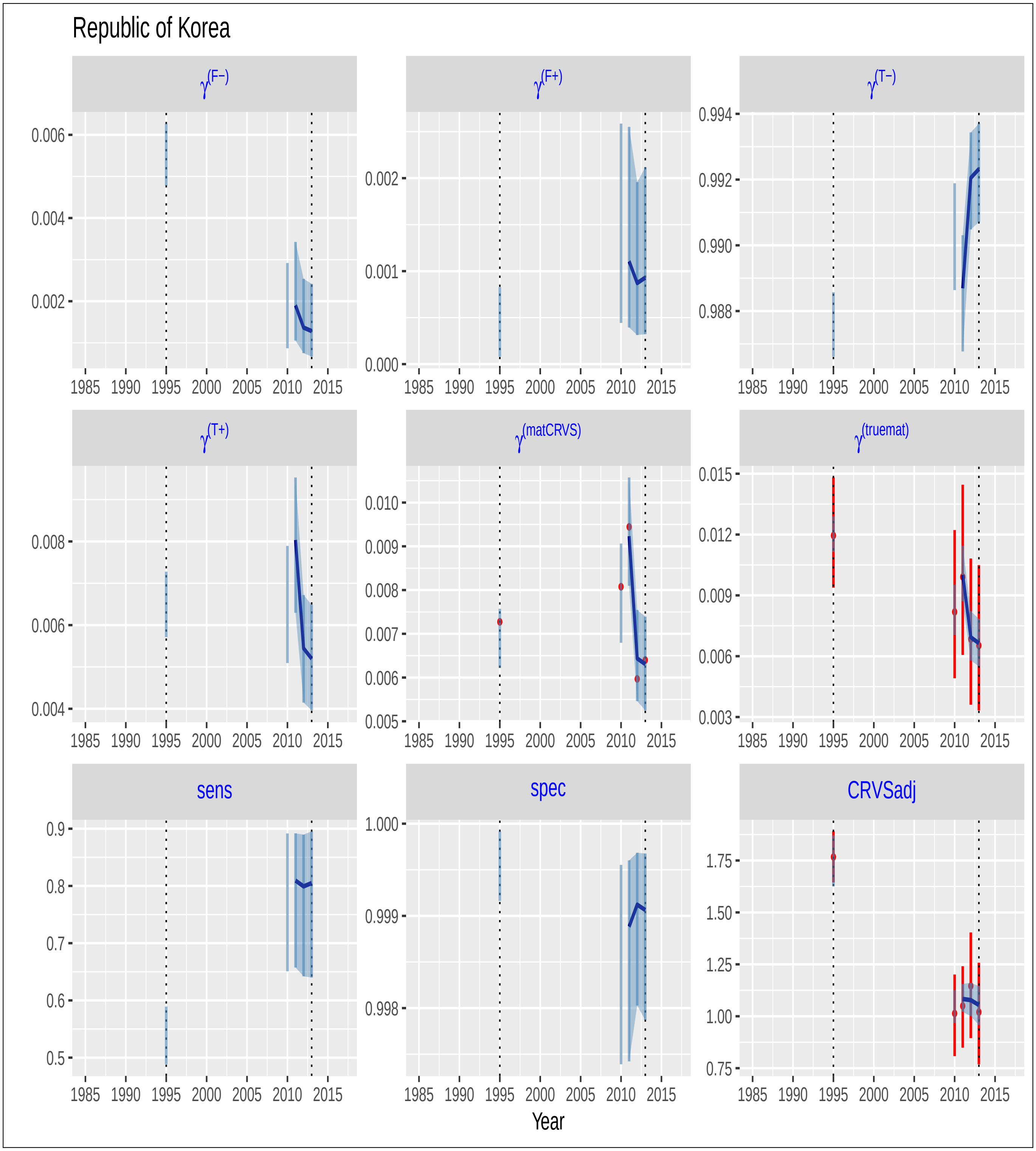

Republic of Korea
γ(F−)
γ(F+)
γ(T−)
γ(T+)
γ(matCRVS)
γ(truemat)
sens
spec
CRVSadj
1985 1990 1995 2000 2005 2010 2015
Year

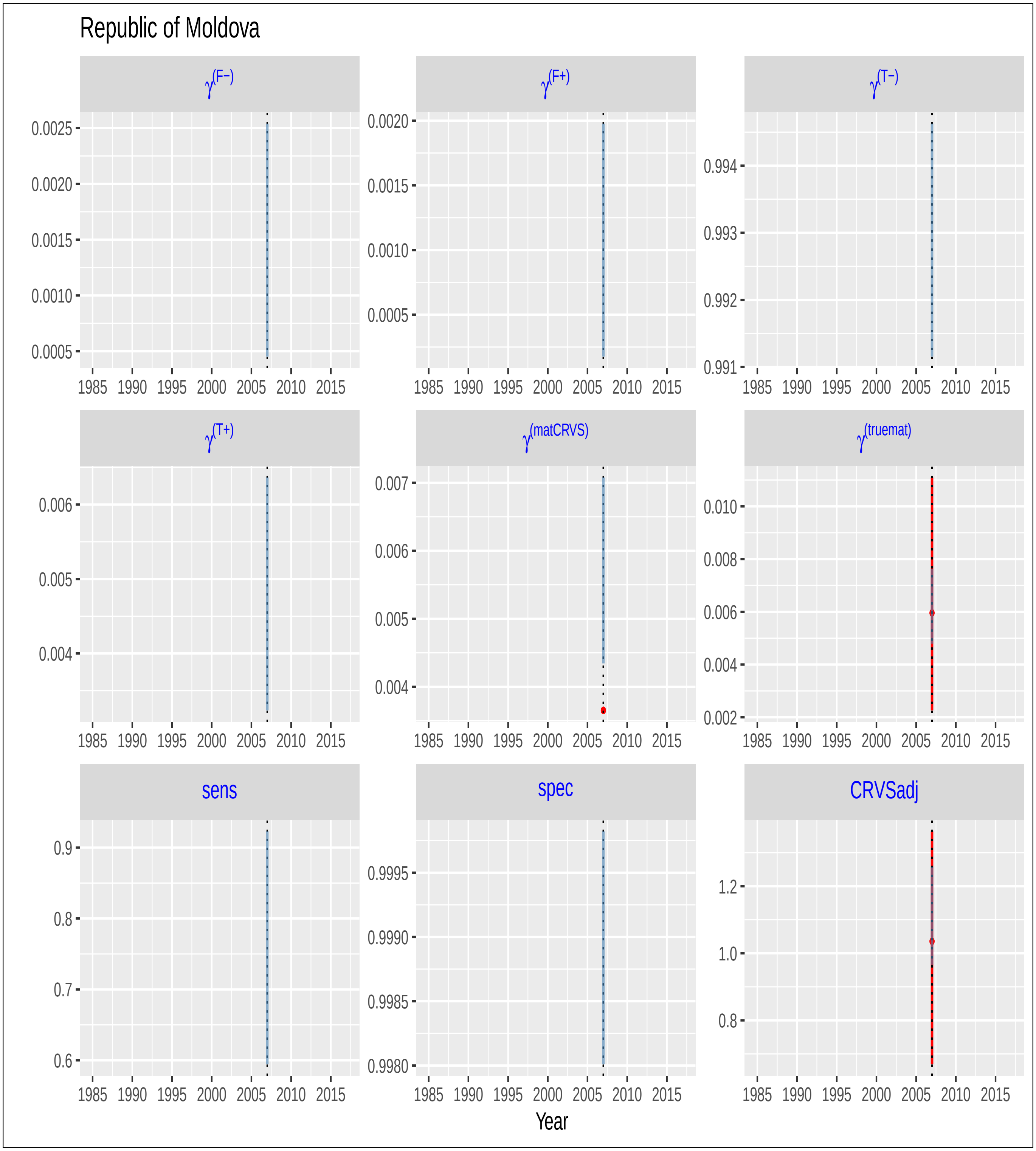
Republic of Moldova
γ(F−)
γ(F+)
γ(T−)
γ(T+)
γ(matCRVS)
γ(truemat)
sens
spec
CRVSadj
Year

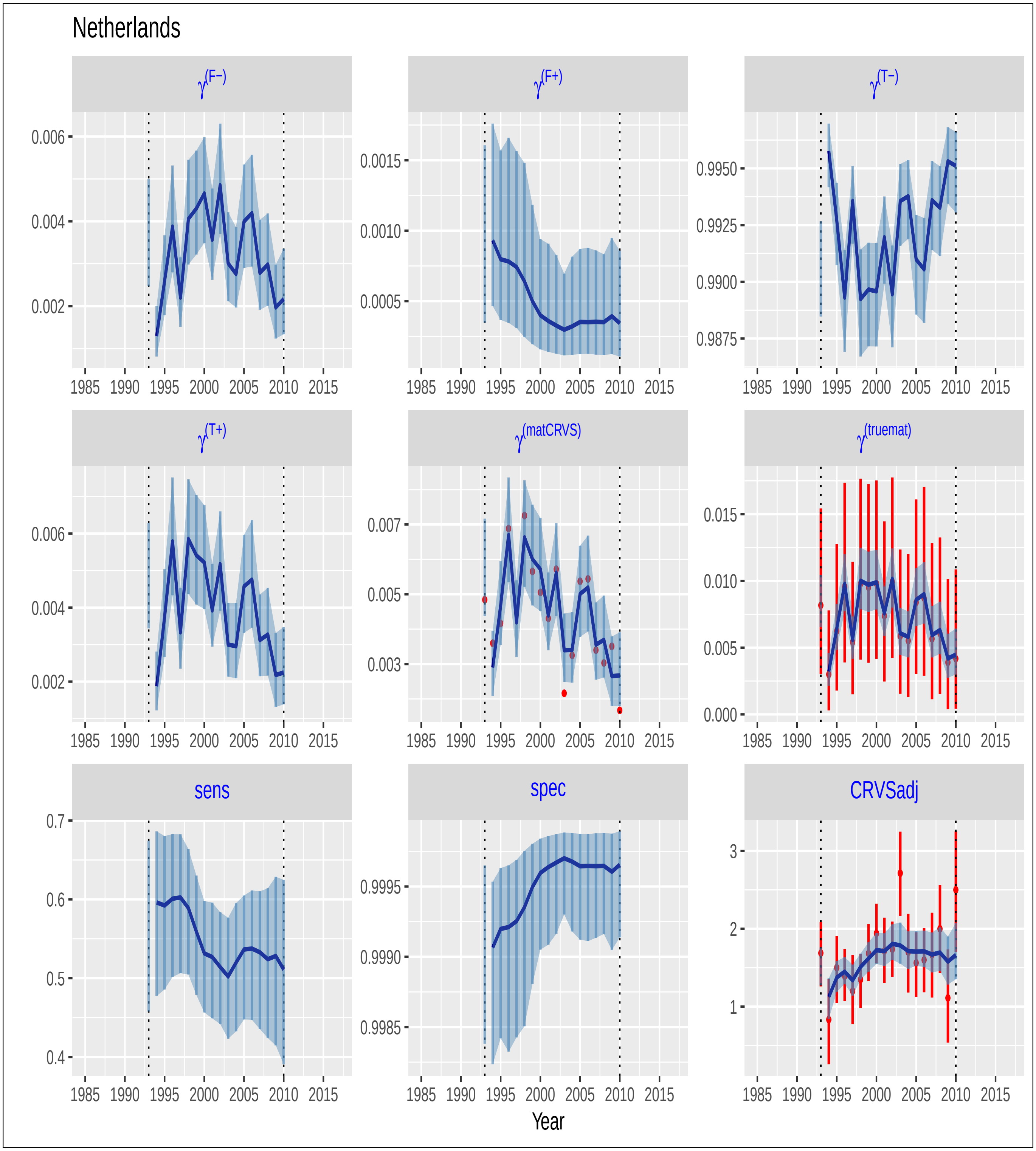

Netherlands
γ(F−)
γ(F+)
γ(T−)
γ(T+)
γ(matCRVS)
γ(truemat)
sens
spec
CRVSadj
0.006
0.004
0.002
0.0015
0.0010
0.0005
0.9950
0.9925
0.9900
0.9875
0.007
0.005
0.003
0.015
0.010
0.005
0.000
0.7
0.6
0.5
0.4
0.9995
0.9990
0.9985
3
2
1
1985 1990 1995 2000 2005 2010 2015
Year

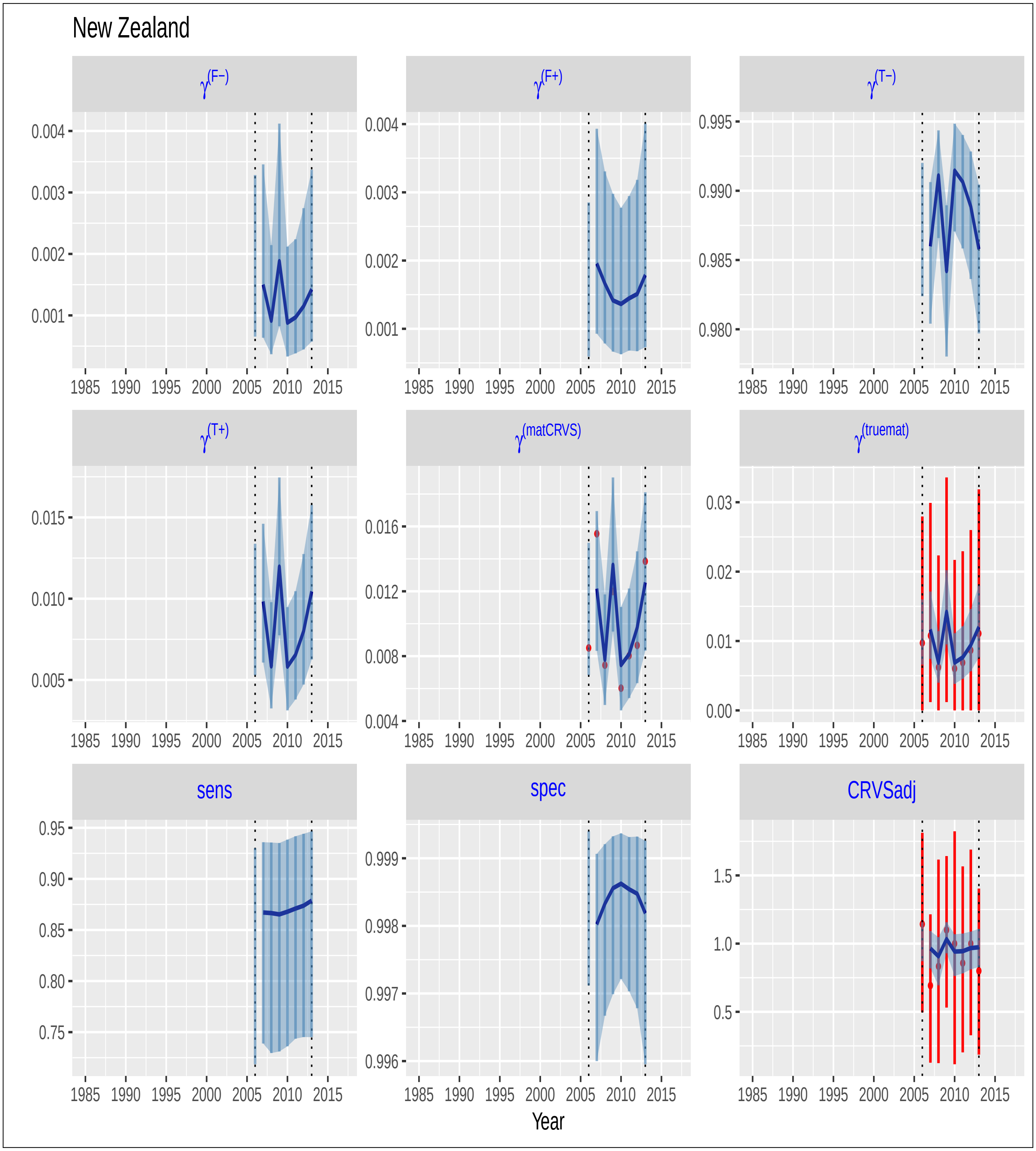

New Zealand
$\gamma^{(F-)}$
$\gamma^{(F+)}$
$\gamma^{(T-)}$
$\gamma^{(T+)}$
$\gamma^{(matCRVS)}$
$\gamma^{(truemat)}$
sens
spec
CRVSadj
0.004
0.003
0.002
0.001
0.995
0.990
0.985
0.980
0.015
0.010
0.005
0.016
0.012
0.008
0.004
0.03
0.02
0.01
0.00
0.95
0.90
0.85
0.80
0.75
0.999
0.998
0.997
0.996
1.5
1.0
0.5
1985 1990 1995 2000 2005 2010 2015
Year

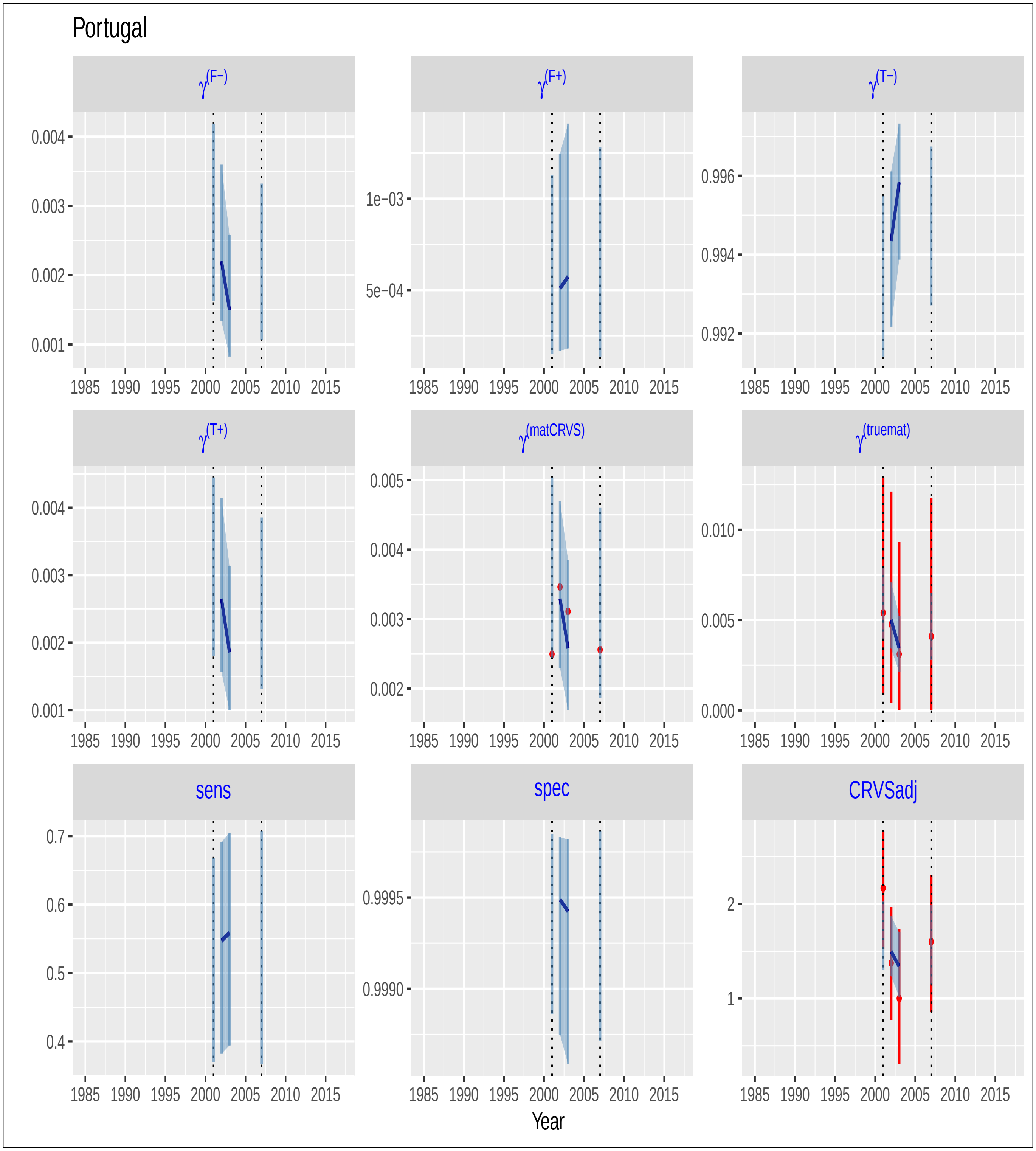

Portugal
γ(F−)
γ(F+)
γ(T−)
γ(T+)
γ(matCRVS)
γ(truemat)
sens
spec
CRVSadj
Year

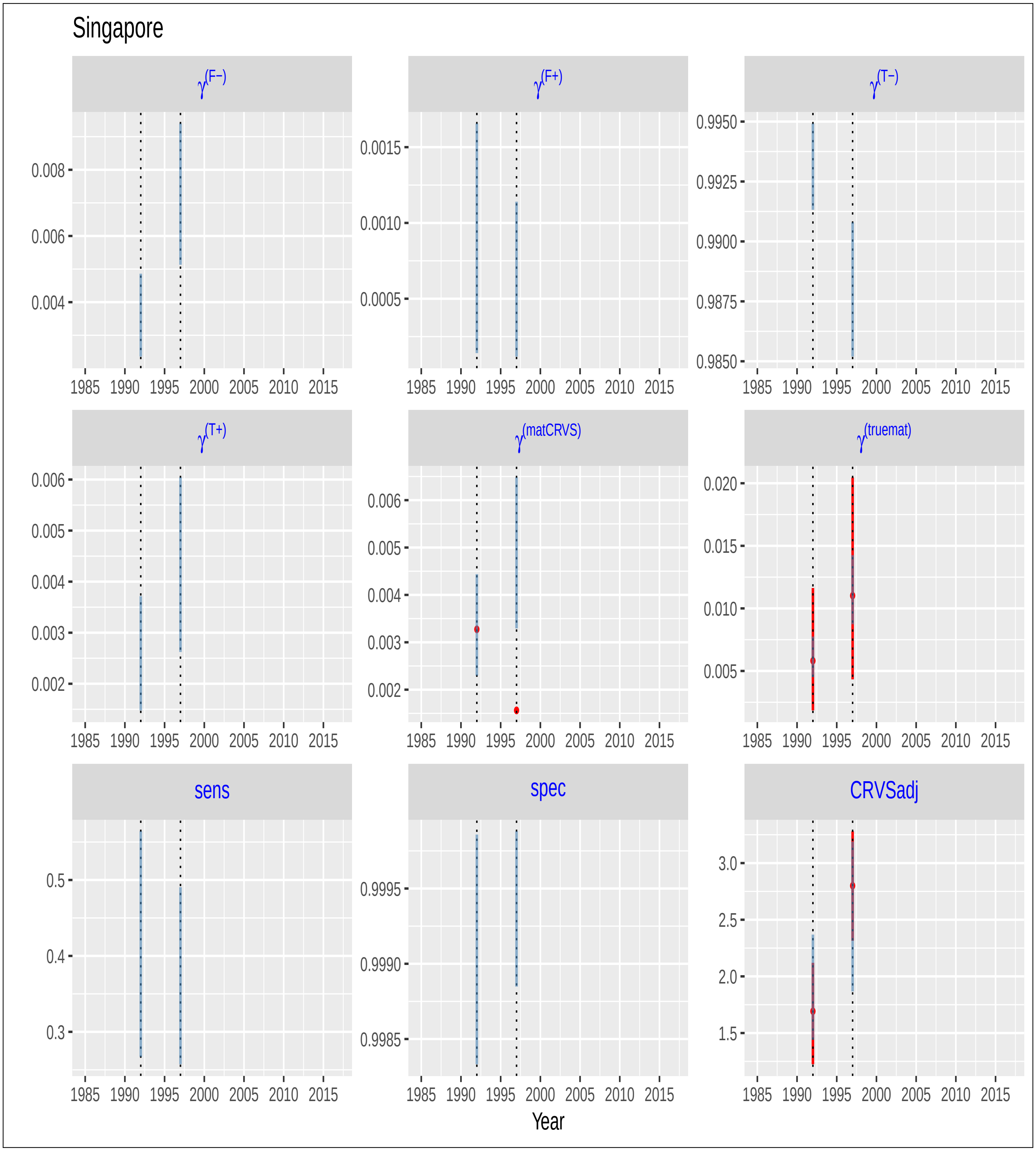

Singapore
γ(F−)
γ(F+)
γ(T−)
γ(T+)
γ(matCRVS)
γ(truemat)
sens
spec
CRVSadj
Year

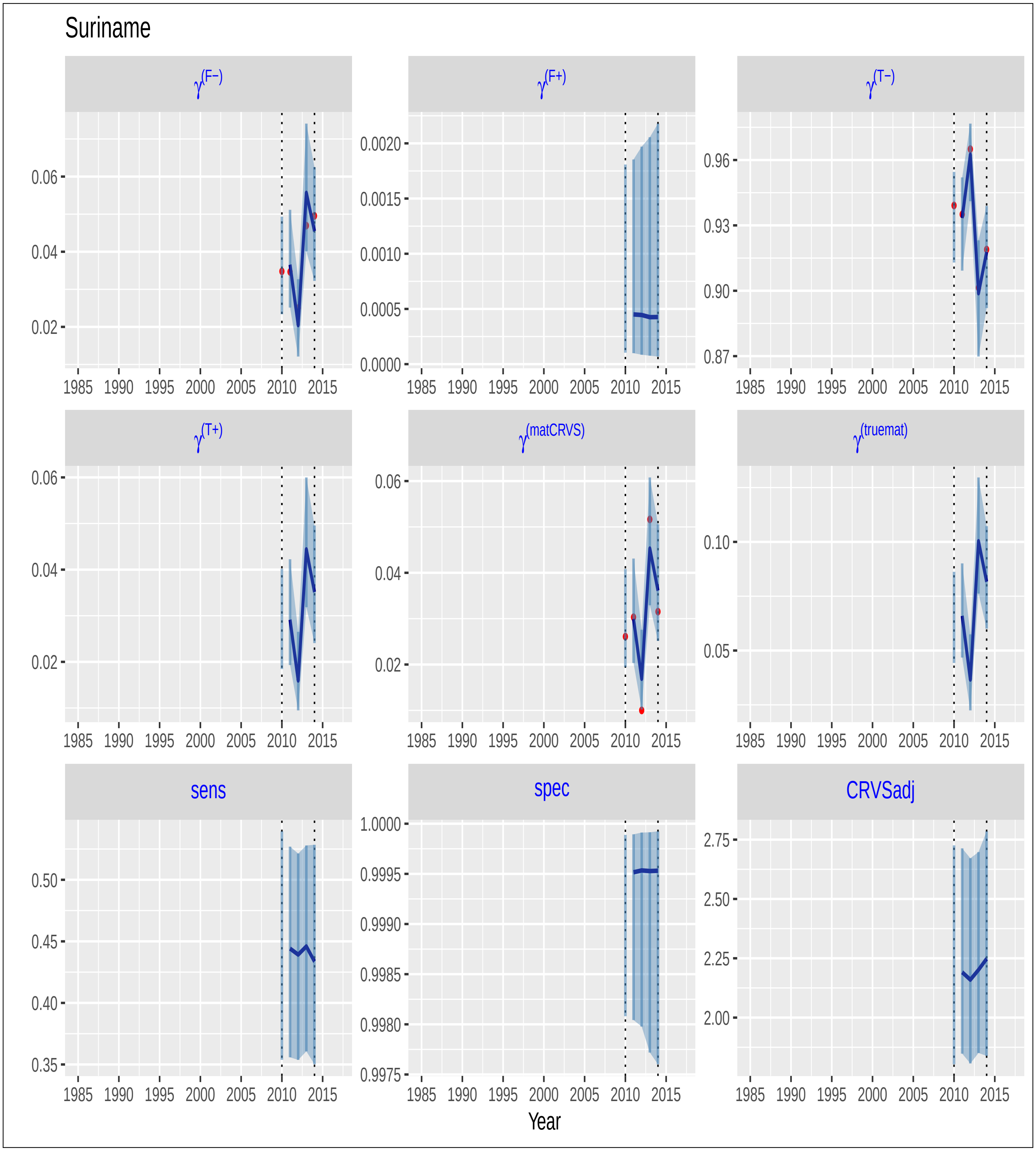

Suriname
γ^(F−)
γ^(F+)
γ^(T−)
γ^(T+)
γ^(matCRVS)
γ^(truemat)
sens
spec
CRVSadj
Year

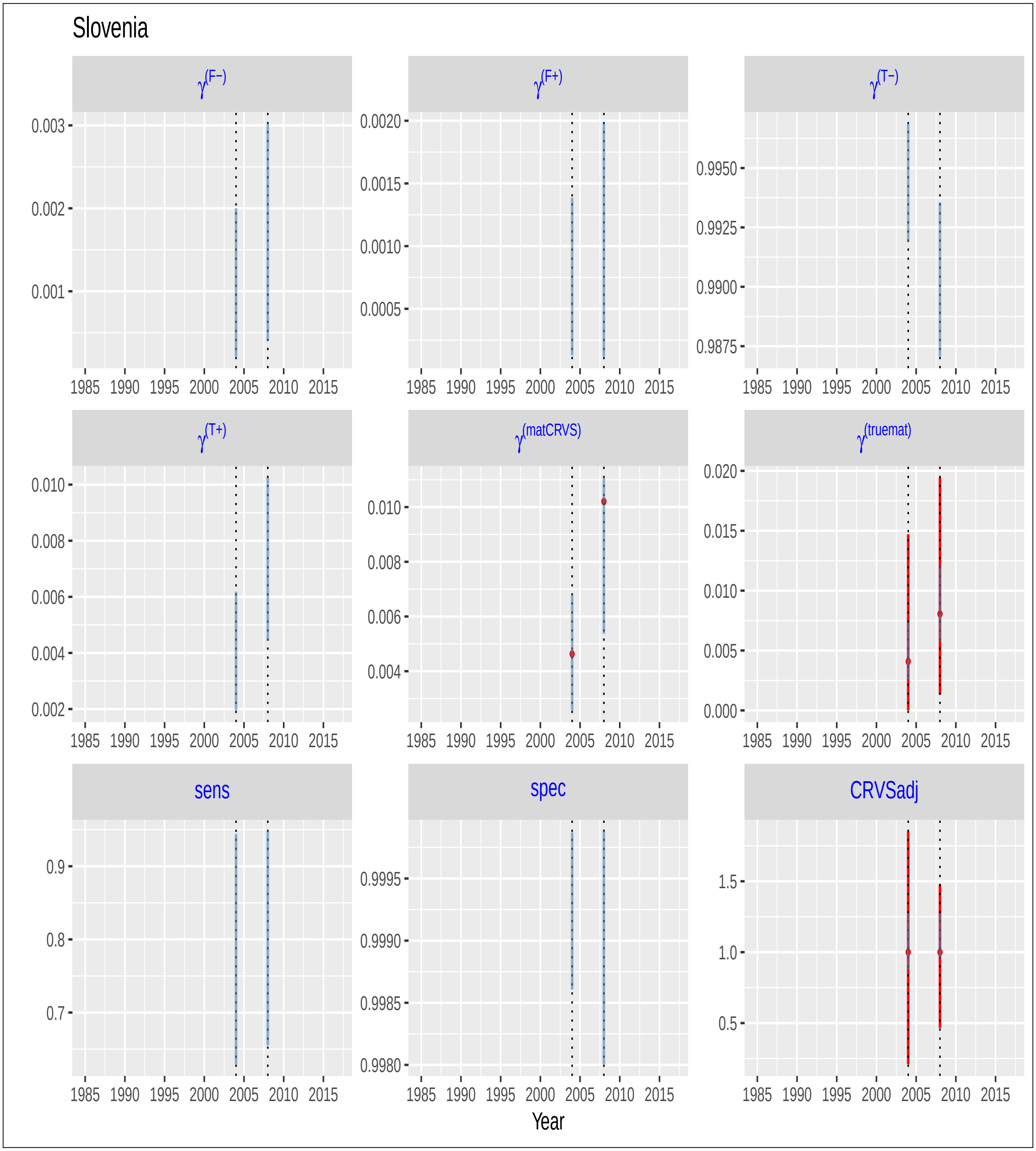
Slovenia
γ(F−)
γ(F+)
γ(T−)
γ(T+)
γ(matCRVS)
γ(truemat)
sens
spec
CRVSadj
Year

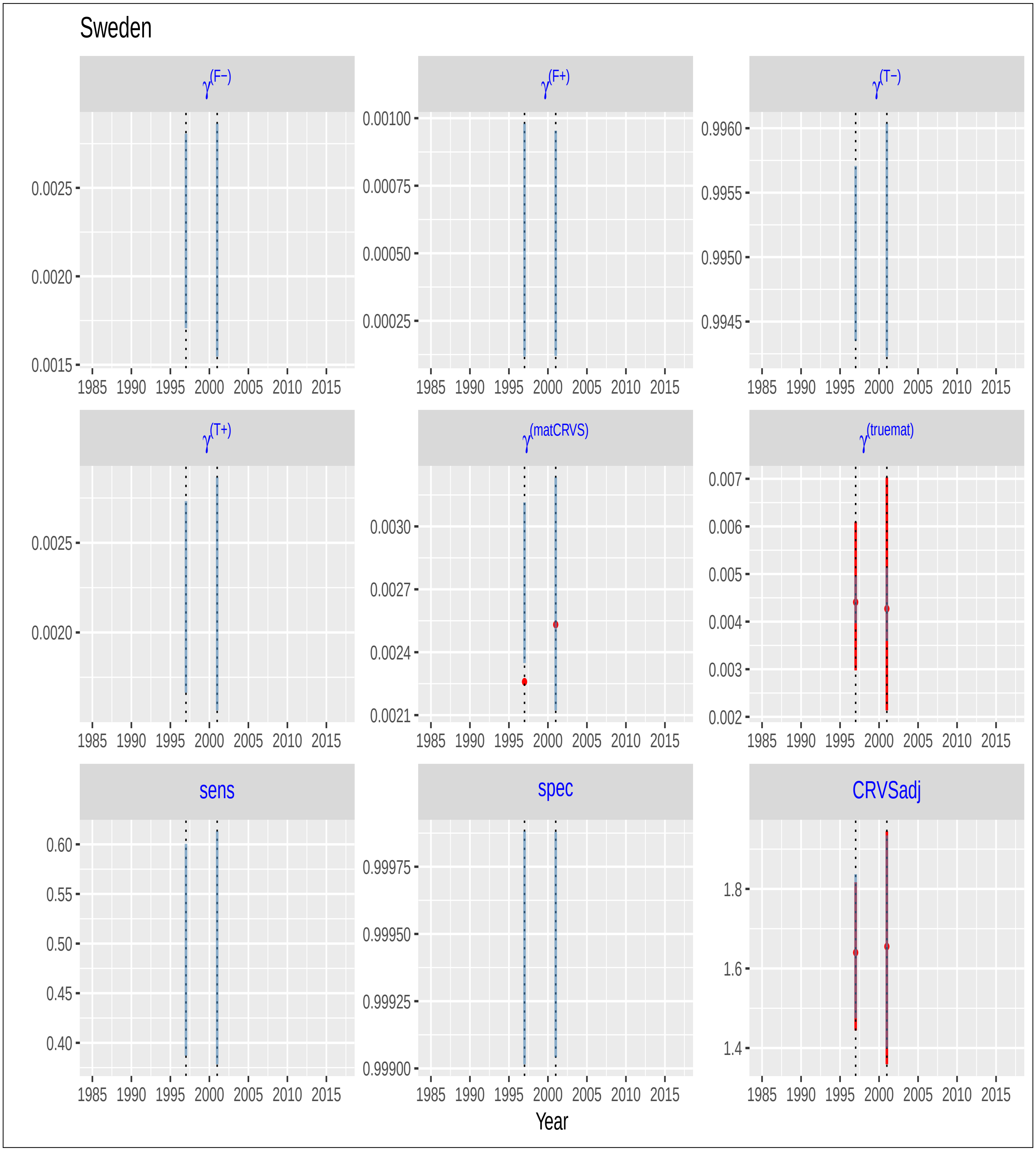

Sweden
γ^(F−)
γ^(F+)
γ^(T−)
γ^(T+)
γ^(matCRVS)
γ^(truemat)
sens
spec
CRVSadj
0.0015
0.0020
0.0025
0.00025
0.00050
0.00075
0.00100
0.9945
0.9950
0.9955
0.9960
0.0021
0.0024
0.0027
0.0030
0.002
0.003
0.004
0.005
0.006
0.007
0.40
0.45
0.50
0.55
0.60
0.99900
0.99925
0.99950
0.99975
1.4
1.6
1.8
1985 1990 1995 2000 2005 2010 2015
Year

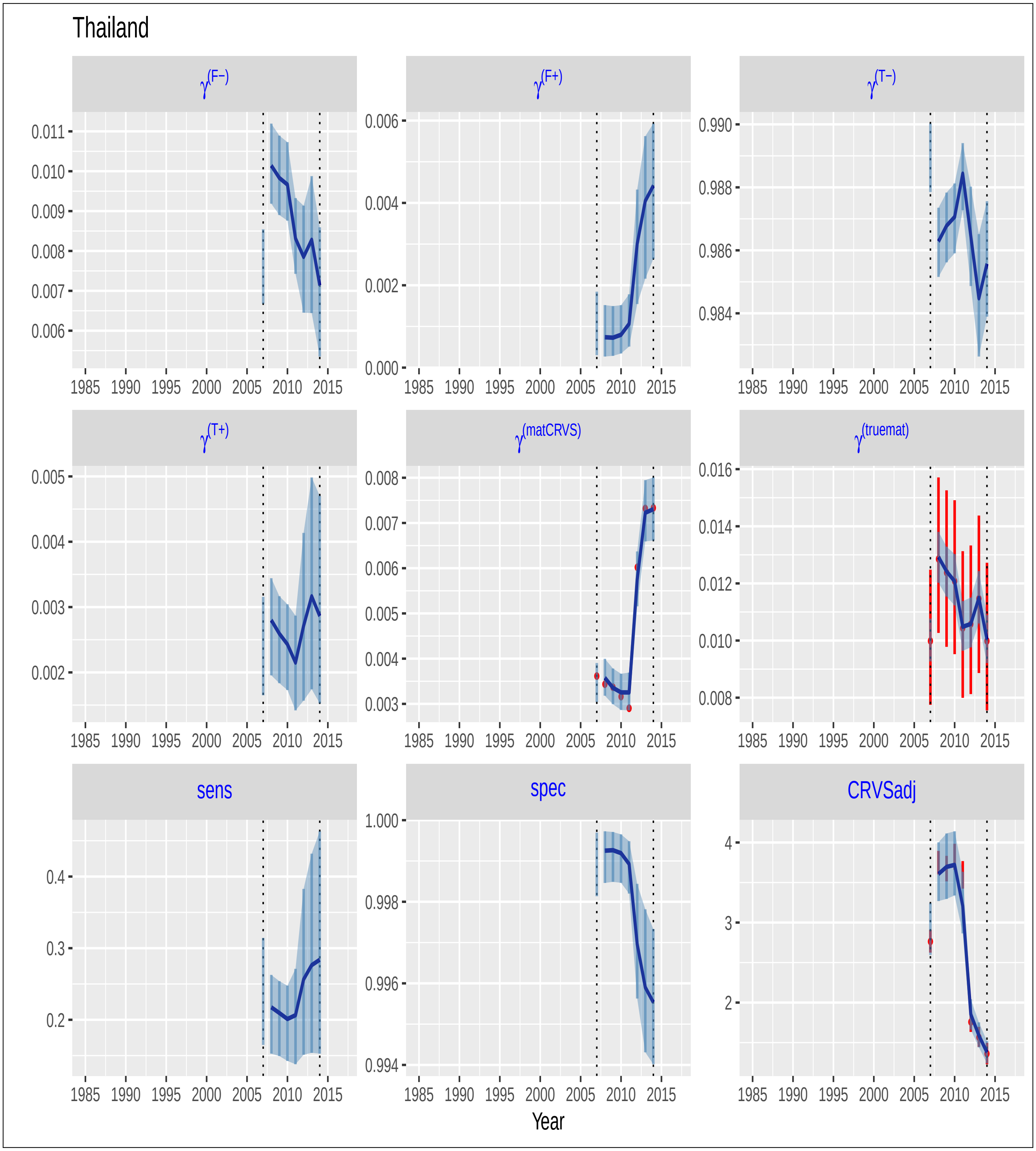

Thailand
γ(F−)
γ(F+)
γ(T−)
γ(T+)
γ(matCRVS)
γ(truemat)
sens
spec
CRVSadj
1985 1990 1995 2000 2005 2010 2015
Year

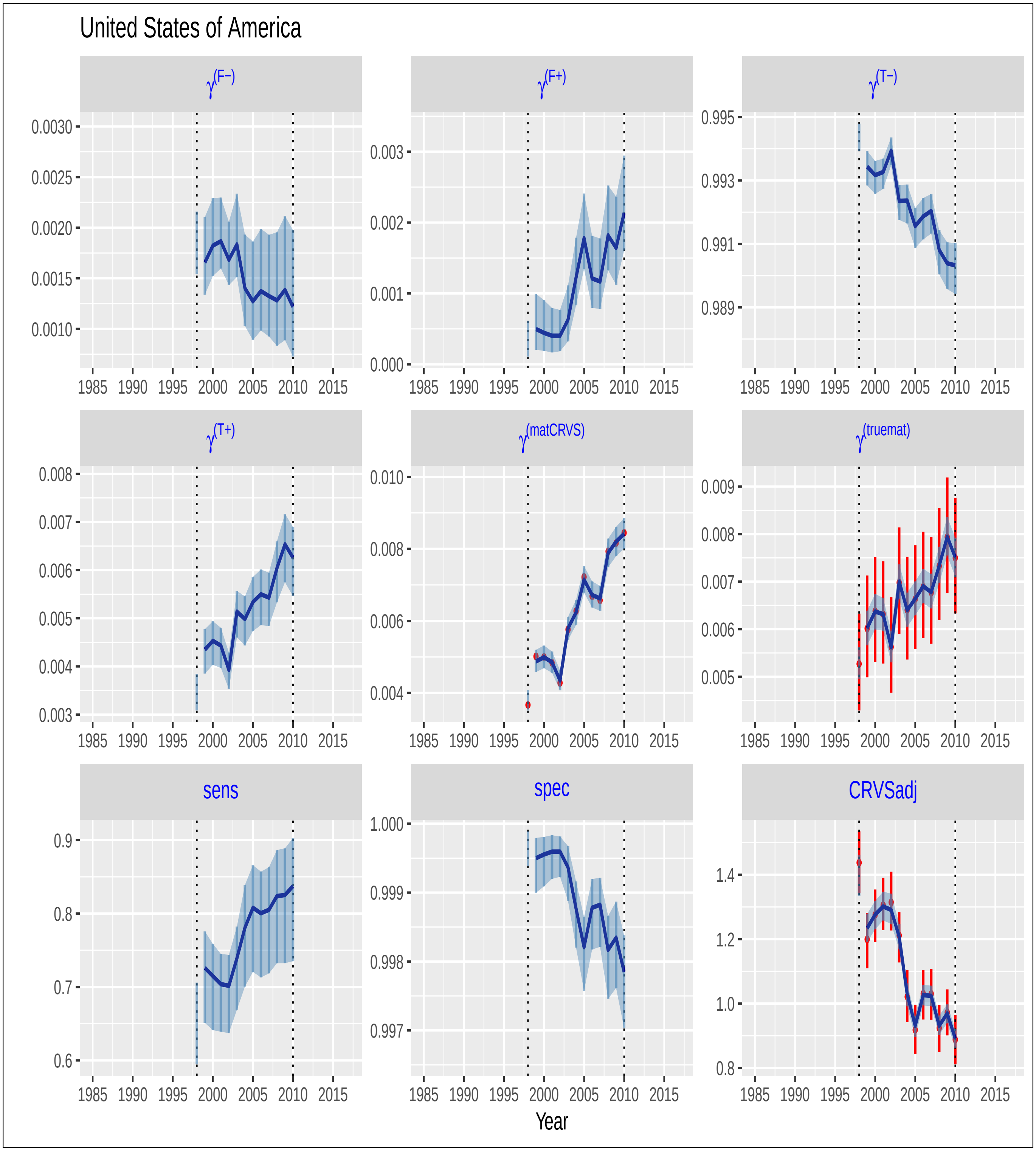

United States of America
γ(F−)
γ(F+)
γ(T−)
γ(T+)
γ(matCRVS)
γ(truemat)
sens
spec
CRVSadj
0.0030
0.0025
0.0020
0.0015
0.0010
0.003
0.002
0.001
0.000
0.995
0.993
0.991
0.989
0.008
0.007
0.006
0.005
0.004
0.003
0.010
0.008
0.006
0.004
0.009
0.008
0.007
0.006
0.005
0.9
0.8
0.7
0.6
1.000
0.999
0.998
0.997
1.4
1.2
1.0
0.8
1985 1990 1995 2000 2005 2010 2015
Year
